# Localization of the CotY and ExsY proteins to the exosporium basal layer of *Bacillus anthracis*

**DOI:** 10.1002/mbo3.1327

**Published:** 2022-10-14

**Authors:** Jorge Durand‐Heredia, George C. Stewart

**Affiliations:** ^1^ Department of Veterinary Pathobiology and Bond Life Sciences Center University of Missouri Columbia Missouri USA

**Keywords:** assembly, exosporium, localization, spore, transcription

## Abstract

Spores are an infectious form of the zoonotic bacterial pathogen, *Bacillus anthracis*. The outermost spore layer is the exosporium, comprised of a basal layer and an external glycoprotein nap layer. The major structural proteins of the inner basal layer are CotY (at the mother cell central pole or bottlecap) and ExsY around the rest of the spore. The basis for the cap or noncap specificity of the CotY and ExsY proteins is currently unknown. We investigated the role of sequence differences between these proteins in localization during exosporium assembly. We found that sequence differences were less important than the timing of expression of the respective genes in the positioning of these inner basal layer structural proteins. Fusion constructs with the fluorescent protein fused at the N‐terminus resulted in poor incorporation whereas fusions at the carboxy terminus of CotY or ExsY resulted in good incorporation. However, complementation studies revealed that fusion constructs, although accurate indicators of protein localization, were not fully functional. A model is presented that explains the localization patterns observed. Bacterial two‐hybrid studies in *Escherichia coli* hosts were used to examine protein–protein interactions with full‐length and truncated proteins. The N‐terminus amino acid sequences of ExsY and CotY appear to be recognized by spore proteins located in the spore interspace, consistent with interactions seen with ExsY and CotY with the interspace proteins CotE and CotO, known to be involved with exosporium attachment.

## Graphical abstract

CotY and ExsY are outer spore layer structural proteins of Bacillus anthracis that are amino acid sequence similar but are found at distinct spore locations. This study found that the timing of protein production, rather than differences in amino acid sequence, is responsible for the site where each protein is incorporated.

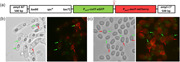

## 1 INTRODUCTION

The genus *Bacillus* is comprised of soil‐dwelling bacteria that utilize sporulation as a survival mechanism. When conditions are unfavorable for growth, such as nutrient limitation, the bacteria undergo a sporulation process to produce spores that are metabolically inert and resistant to a variety of environmental insults including heat and desiccation. The outer surface of the spore consists of glycoproteins. With *Bacillus subtilis*, this layer is referred to as the crust and is associated with the outer spore coat (Imamura et al., 2011; McKenney et al., 2010). Certain *Bacillus* species produce spores that possess an outer spore layer, the exosporium. It is a deformable protein shell that is separated from the spore coat by the interspace layer (Giorno et al., 2009). The exosporium consists of a basal layer and a hairlike nap layer containing the BclA collagen‐like glycoprotein (Stewart, 2015; Sylvestre et al., 2002; Sylvestre et al., 2005). The basal layer of the *B. anthracis* exosporium is approximately 12–16 nm thick and appears to be comprised of two, approximately 5‐nm‐thick sublayers (Rodenburg et al., 2014). The exosporium is thought to be a semi‐permeable barrier that excludes potentially harmful large molecules such as antibodies and hydrolytic enzymes, but permits the passage of small molecules such as germinants (Ball et al., 2008; Gerhardt & Black, 1961). The exosporium also confers hydrophobic properties on the spore, likely playing a role in persistence of spores in soil environments (Williams et al., 2013). However, with the zoonotic pathogen *Bacillus anthracis*, the exosporium is also the site of early interactions between the infectious spores and macrophages and dendritic cells of the host innate immune system during the initial stages of the infectious process (Bozue et al., 2007; Brahmbhatt et al., 2007; Oliva et al., 2009).

Synthesis of the exosporium initiates early in the sporulation process, at the stage of engulfment of the forespore by the mother cell. Before engulfment is complete, a thin layer, the bottlecap (or simply the cap) is evident at the mother cell central pole of the developing spore. Later in the sporulation process, coincident with spore coat assembly, the exosporium basal layer is assembled from the cap toward the noncap pole. Three important structural proteins of the basal layer are BxpB, CotY, and ExsY (Boydston et al., 2006; Johnson et al., 2006; Lablaine et al., 2021; Steichen et al., 2005; Sylvestre et al., 2005; Terry et al., 2017). *B. anthracis* mutants deleted for the *exsY* determinant produce spores possessing only the bottlecap portion of the exosporium (Boydston et al., 2006). This corresponds to approximately 25% of the exosporium at the mother cell central pole of the developing spore. The *exsY* mutants do not produce the noncap portion of the exosporium, indicating that ExsY is a major structural protein of the noncap basal layer. Mutants deleted for *cotY* produce an intact exosporium, but with altered assembly kinetics. The CotY protein is a structural component of the cap, and *cotY* mutants fail to produce the cap in the early stages of sporulation (Boone et al., 2018; Lablaine et al., 2021; Terry et al., 2017).

The exosporium is assembled at the time that the spore coat is assembled (Boone et al., 2018). During the early stages of assembly, the exosporium closely abuts the spore coat layer. Later, the exosporium separates from the spore coat, creating an electron translucent space between the two layers called the interspace (Giorno et al., 2009). During assembly, the exosporium is anchored to the spore coat. Mature spores from mutants lacking CotE or CotO proteins lack the exosporium layer (Boone et al., 2018; Giorno et al., 2007; Lablaine et al., 2021). CotE and *cotO* mutants produce the exosporium in sheets in the mother cell cytoplasm adjacent to the bottlecap pole of the spore (Boone et al., 2018; Giorno et al., 2007), indicating that assembly of the exosporium can occur in the absence of these anchoring proteins, but cannot encapsulate the developing spore and is lost following mother cell lysis. It is noteworthy that CotO has a role during crust assembly in *B. subtilis*, promoting spore encasement (Shuster et al., 2019). Mutant spores lacking the ExsA and ExsB proteins have also been reported to have exosporium attachment deficient phenotypes (Bailey‐Smith et al., 2005; McPherson et al., 2010). However, loss of ExsA in *Bacillus cereus* also resulted in major defects in spore coat assembly, and the effects on exosporium attachment may be indirect effects due to loss of CotE or CotO.

This study is focused on the assembly of the CotY and ExsY basal layer proteins and factors which influence their correct localization within the exosporium. It also highlights the advantages, and limitations, of visualizing exosporium assembly utilizing fluorescent fusion proteins.

## 2 MATERIALS AND METHODS

### 2.1 Bacterial strains, plasmids, and culture conditions

Bacterial strains and plasmids are listed in Table A1. All *Escherichia coli* strains were cultivated using a lysogeny broth (LB) medium (0.5% yeast extract, 1% tryptone, and 1% NaCl). *B. anthracis* was grown using Brain Heart Infusion broth (BHI, Difco). Agar plates were made by the addition of agar at a concentration of 1.5% (w/v). Nutrient broth and agar (Oxoid) were used for sporulation. Antibiotics where needed were added to the following final concentrations: ampicillin (100 μg/ml), chloramphenicol (10 μg/ml), kanamycin (25 μg/ml), and spectinomycin (100 μg/ml).

### 2.2 DNA purification

The Wizard SV miniprep kit (Promega) was used to isolate plasmid DNA. For *B. anthracis*, the pellets from 5 ml cultures were frozen at −80°C overnight and thawed at 37°C before DNA extraction. Genomic DNA was isolated using the Wizard Genomic DNA purification kit (Promega). For *B. anthracis*, the cell pellets were frozen at −80°C overnight and thawed at 37°C before DNA extraction.

### 2.3 Construction of complementation and expression plasmids

Expression of *Bacillus* proteins was accomplished by the introduction of the gene with its native or heterologous promoter into the shuttle plasmids pMK4 (Sullivan et al., 1984) or pHPS2 (Thompson et al., 2011). The shuttle plasmid pHPS2 is a derivative of pHP13, it is a relatively low copy number replicon with a copy number of ~5 in *B. subtilis* hosts (Haima et al., 1987). The *cotY* and *exsY* chimeras as well as the promoter exchanged constructs were constructed by splicing using overlapping extension polymerase chain reaction (PCR) (Horton et al., 1993). All constructs were nucleotide sequence verified before transformation into *B. anthracis* strains.

### 2.4 Construction of the gene fusions to *mcherry* or *egfp*

The fluorescent reporter fusions were generated by PCR amplifying the *B. anthracis* genes using primers with a 5′ *Sac*I or *Pst*I restriction site upstream of the native promoter element and a 3′ *Nhe*I site immediately before the termination codon of the open reading frame. *Sac*I/*Pst*I and *Nhe*I digested DNA fragments were then cloned into identically digested pDG4100 (for mCherry fusions) or pDG4099 (for eGFP fusions) and transformed into *E. coli* strain DH5α. Clones with plasmids of the correct size and restriction endonuclease pattern were identified and their insert sequence verified by nucleotide sequence analysis. The plasmids were then transformed into *E. coli* GM48, plasmid DNA isolated and electroporated into *B. anthracis* Sterne.

### 2.5 Generation of *B. anthracis* deletion mutants

The gene deletion mutants were generated by PCR amplifying and cloning 1‐kb sequences upstream and downstream of the *cotY* or *exsY* open reading frame. The upstream and downstream fragments were fused by splicing overlap exchange PCR. The resulting 2‐kb fragment was cloned into *Sal*I‐digested and alkaline phosphatase‐treated pGS4294. A spectinomycin resistance cassette flanked by *lox66*/*lox71* sites (Lambert et al., 2007) was inserted into the *Bam*HI site at the position of the deleted gene. Sequence‐verified plasmids were isolated from *E. coli* GM48 and electroporated into *B. anthracis* Sterne and incubated at 30°C. Colonies exhibiting spectinomycin resistance were inoculated onto spectinomycin and erythromycin (the pGS4294 vector‐encoded resistance) plates to ensure no spontaneous spectinomycin resistant colonies arose and that cells from the colony harbored the allele‐replacement plasmid. Following confirmation of both Spc^R^ and Ery^R^ of the transformants, the resulting *B. anthracis* clones were inoculated into 10 ml of BHI broth containing spectinomycin and grown overnight at 42°C with shaking. Thirty microliters of the culture was then plated on a BHI agar + spectinomycin plate and streaked for isolation of single colonies and grown overnight at 37°C. Larger colonies were selected with this semi‐selective incubation temperature. This process of growing an overnight liquid culture at 42°C and plating was repeated until PCR analysis of DNA from the clone using primers flanking the gene to be deleted gave only the DNA fragment size corresponding to the deletion allele. Sequence analysis of the PCR fragment confirmed the deletion.

To create double or triple mutants, the deletion mutant strain was transformed with plasmid pGS4080, a segregationally unstable plasmid encoding the *cre* recombinase expressed from the *spac* promoter. Chloramphenicol‐resistant transformants were selected and then subcultured on TSA plates lacking chloramphenicol at 37°C. The resulting colonies were screened for sensitivity to chloramphenicol and spectinomycin. Clones with the correct antibiotic‐resistance profile were selected, genomic DNA extracted, and screened by PCR and DNA sequencing to confirm the deletion of the *lox*‐flanked spectinomycin resistance cassette. The deletion clone was then utilized to create a deletion at another locus.

### 2.6 Single copy ectopic insertion into the *B. anthracis* chromosome

The *amyS* allele replacement vector, pGS6328, was constructed using the temperature‐sensitive shuttle plasmid pGS4294, comprised of pUC18 and a temperature‐sensitive derivative of the staphylococcal plasmid pE194. A DNA fragment consisting of the first and last 500 bp of the *B. anthracis amyS* orf (bas3291) with a unique *Sal*I site in the center was inserted. Adjacent to the truncated *amyS* allele is a spectinomycin resistance cassette flanked by *lox66* and *lox71 loxP* sites, to facilitate Cre‐mediated removal of the resistance cassette, if needed. For the pJD6434 plasmid, the two inserted fusion genes were separated by the *cotY* transcription terminator sequence (**TAA**AACTAAATAATGAGCTAAGCATGGATTGGGTGGCAGAATTATCTGCCACCCAATCCATGCTTAACGAGTATTATTAT, with the stem sequences underlined and the *cotY* stop codon shown in bold) to prevent bleeding through transcription from the upstream *cotY* promoter. Electroporation and allele replacement mutagenesis were conducted as previously described (Hermanas et al., 2021). All plasmids used for electroporation were passed through *E. coli* GM48 to produce DNA lacking the Dam methylation pattern. Colonies exhibiting spectinomycin resistance were inoculated onto spectinomycin and erythromycin (the pGS4294 vector‐encoded resistance) plates to ensure no spontaneous spectinomycin resistant colonies arose and that cells from the colony harbored the allele‐replacement plasmid. Following confirmation of both Spc^R^ and Ery^R^ of the transformants, the resulting *B. anthracis* clones were inoculated into 10 ml of BHI broth containing spectinomycin and grown overnight at 42°C with shaking. Thirty microliters of the culture was then plated on a BHI agar + spectinomycin plate and streaked for isolation of single colonies and grown overnight at 37°C. Larger colonies were selected with this semi‐selective incubation temperature. This process of growing an overnight liquid culture at 42°C and plating was repeated until PCR analysis of DNA from the clone using primers flanking the gene to be deleted gave the DNA fragment size corresponding to the deletion allele. Sequence analysis of the PCR fragment confirmed the allele replacement.

### 2.7 Production of spores

Cells from the BHI broth culture were swab inoculated with the *B. anthracis* strain onto the surface of 150 mm × 15 mm Oxoid nutrient agar plates with the appropriate antibiotics. The cultures were incubated at 30°C for 5 days. The surface layer of bacterial growth was harvested with a sterile cotton swab and the spores dispersed into phosphate‐buffered saline (PBS). The spores were harvested by centrifugation at 15,000 rpm and the upper pellet layer containing lysed cell debris was removed by flushing and aspiration and then discarded. The spore pellet was then resuspended by vortex mixing in PBS and the process was repeated until there was no evidence of vegetative cells or cell debris present. Spores were resuspended in PBS and stored at 4°C.

### 2.8 Immunolabeling of spores

Ten milligrams of spores were resuspended in 750 μl SuperBlock Blocking buffer (Thermo Scientific) and incubated for at least 20 min at room temperature. The spores were then harvested by centrifugation and the spore pellet was resuspended in 250 μl SuperBlock blocking buffer with 1 μl primary antibody and incubated at room temperature for 20 min (with mixing every 5 min). Rabbit polyclonal anti‐rBclA antibodies were used (Thompson et al., 2007). Following incubation with the primary antibody, the spores were harvested by centrifugation and washed with 750 μl of SuperBlock blocking buffer. The pellet was then resuspended in 250 μl of SuperBlock Blocking buffer with secondary antibody conjugate (1:250 goat anti‐rabbit IgG‐Alexa Fluor 568; Invitrogen). The spores were incubated at room temperature for 20 min, pelleted, and washed with 750 μl SuperBlock blocking buffer, followed by three washes with 750 μl PBS, and finally resuspended in 250 μl PBS. The spores were examined by epi‐fluorescence microscopy using a Nikon E600 epifluorescence microscope using the mCherry filter set.

### 2.9 Protein interaction analysis by the bacterial two‐hybrid method

The procedure of Karimova et al. (2017) was used. Plasmids pKT25, pKNT25, pUT18, and pUT18C were utilized, thus obtaining hybrid proteins with the T18 or T25 domains of adenylate cyclase on their N‐ or C‐terminus. All plasmid constructs (Table A1) were verified by restriction analysis and by DNA sequencing. The compatible recombinant plasmid pairs were cotransformed into *E. coli* BTH101 competent cells. Transformants were selected on LB plates supplemented with ampicillin (100 μg/ml) plus kanamycin (50 μg/ml) and were cultivated at 30°C for 48 h. The plasmids pKT25‐zip and pUT18C‐zip served as positive control and a pairing of ExsY with the pXO1‐encoded AW20_5669 proteins was used as the negative control. β‐galactosidase assays were performed as described by Schaefer et al. (2016) utilizing PopCulture^TM^ reagent as the lysis reagent (Millipore Sigma) and a BioTek Synergy 96‐well microplate reader.

## 3 RESULTS

### 3.1 CotY and ExsY are assembled at distinct sites in the *B. anthracis* exosporium basal layer

**Figure 1:**
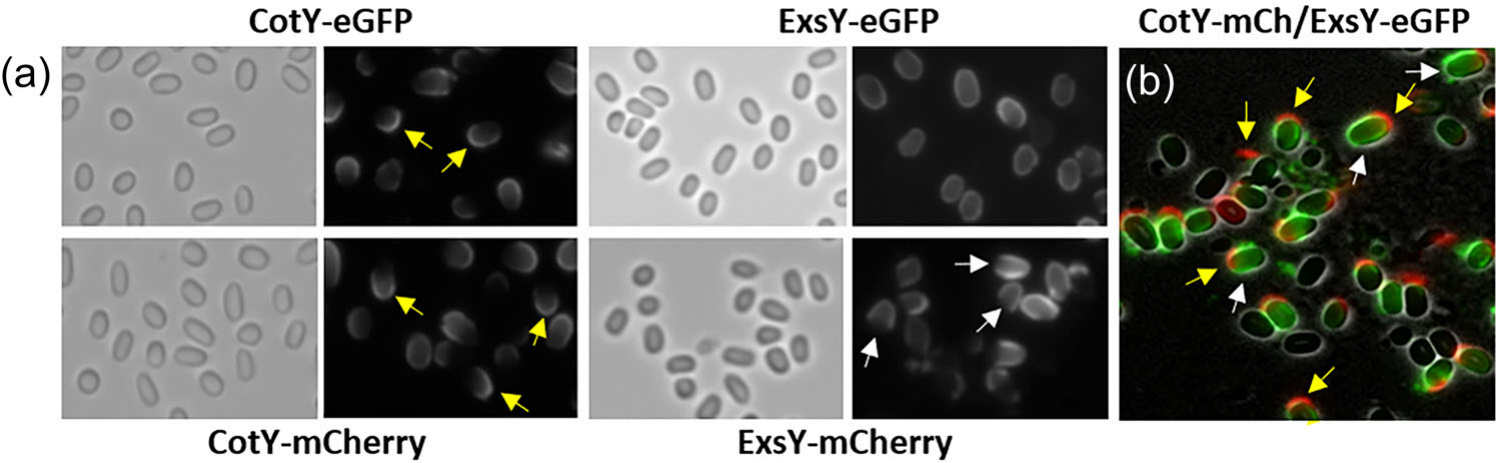
(a) Localization of fluorescent fusions of CotY and ExsY in spores of *Bacillus anthracis* Sterne bearing the indicated plasmid‐encoded fusion genes. The left panel of each pair is the brightfield image and the right panel contains the corresponding fluorescence image. (b) Fluorescent image of spores of *B. anthracis* Sterne expressing both CotY‐mCherry and ExsY‐eGFP. Yellow arrows indicate examples of CotY fusion protein localization at one pole of the spore. White arrows indicate examples of ExsY fusion fluorescence at the noncap portion of the exosporium.

**Figure 2:**
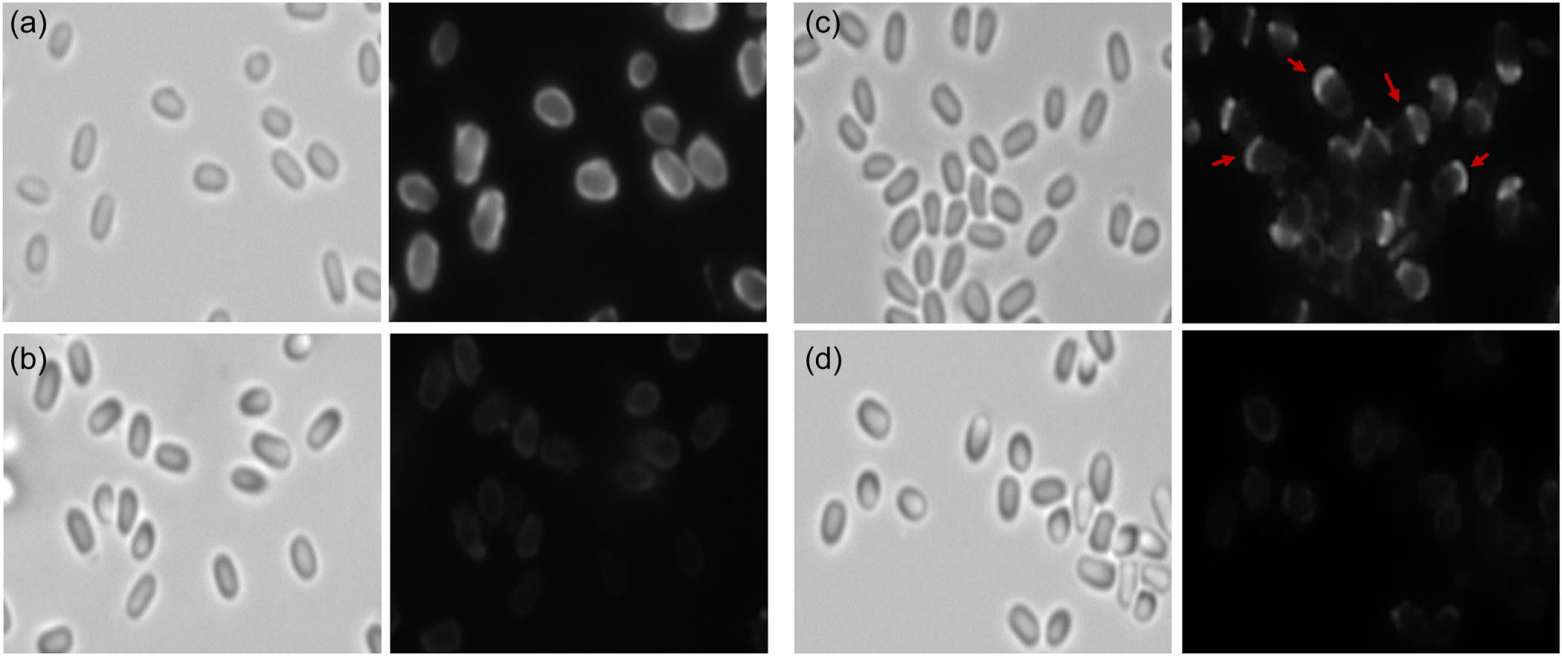
Localization pattern of CotY‐mCherry and ExsY‐mCherry by fluorescence microscopy in mutant host strains. The left panel of each pair is the brightfield image and the right panel contains the corresponding fluorescence image. (a) Δ*cotY* pHPS2‐*cotY‐mcherry*, (b) Δ*cotY* Δ*exsY* pHPS2‐*cotY‐mcherry*, (c) Δ*exsY* pHPS2‐*exsY‐mcherry* and (d) Δ*cotY* Δ*exsY* pHPS2‐*exsY‐mcherry*. Red arrows denote examples of fluorescence at only one pole of the spore. The faint fluorescence in panels b and d are better visualized if the image is enlarged when viewed.

**Figure 3:**
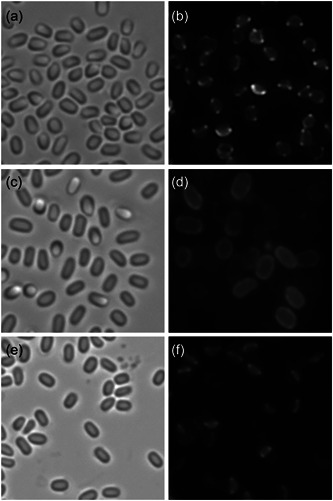
Failure of the *exsY‐mcherry* determinant to complement *ΔexsY*. Bright‐field images of spores (a, c, e), anti‐BclA fluorescence (b, d), and ExsY‐mCherry fluorescence (f) of Sterne *ΔexsY* (a, b), *ΔexsY* pHPS2‐*exsY* (c, d), and *ΔexsY* pHPS2‐*exsY‐mcherry* (e, f).

**Figure 4:**
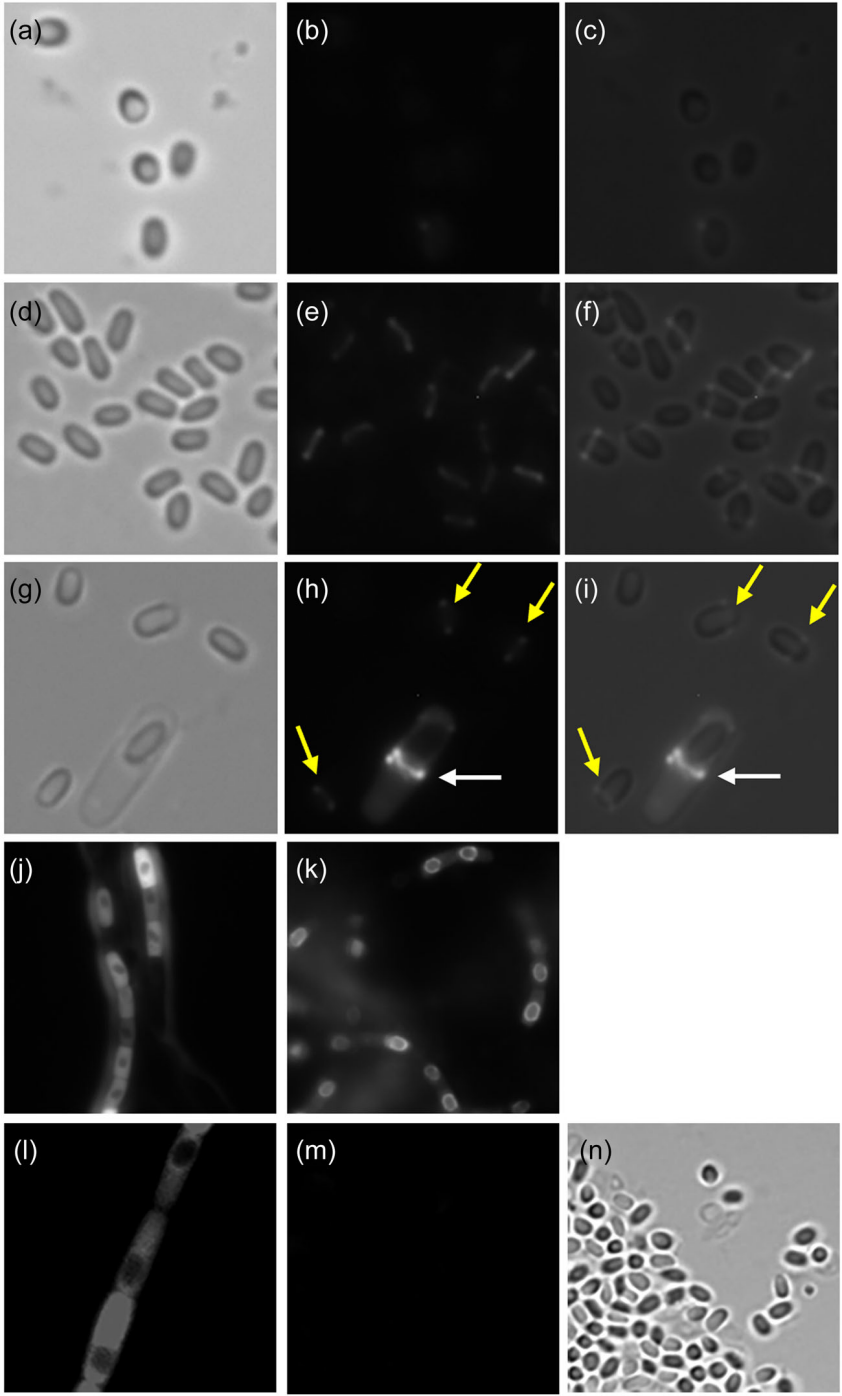
Localization pattern of mCherry‐ExsY by fluorescence microscopy. Bright‐field (a, d, g, n), fluorescence images (b, e, h, j, k, l, m), and merged images (c, f, i) are shown. (a–c) Sterne pHPS2‐*mcherry‐exsY* and (d–i) Δ*exsY* pHPS2‐*mcherry‐exsY*. (g–i) A sporulating cell which shows the enhanced fluorescence relative to that observed with released spores. (j) Sporulating cells expressing mCherry‐ExsY and (k) shows sporulating cells expressing ExsY‐mCherry at the same stage of sporulation. (l) shows sporulating cells expressing unfused mCherry expressed from the exosporium gene *bclA* promoter, (m) is a fluorescence image of mature spores from the (n) field, and (n) is the bright field image of (m). Yellow arrows denote examples of the weak fluorescence at the bottlecap margin of the exosporium of released spores and the white arrows indicate stronger fluorescence at the mother cell central pole of the sporulating cell.

**Figure 5:**
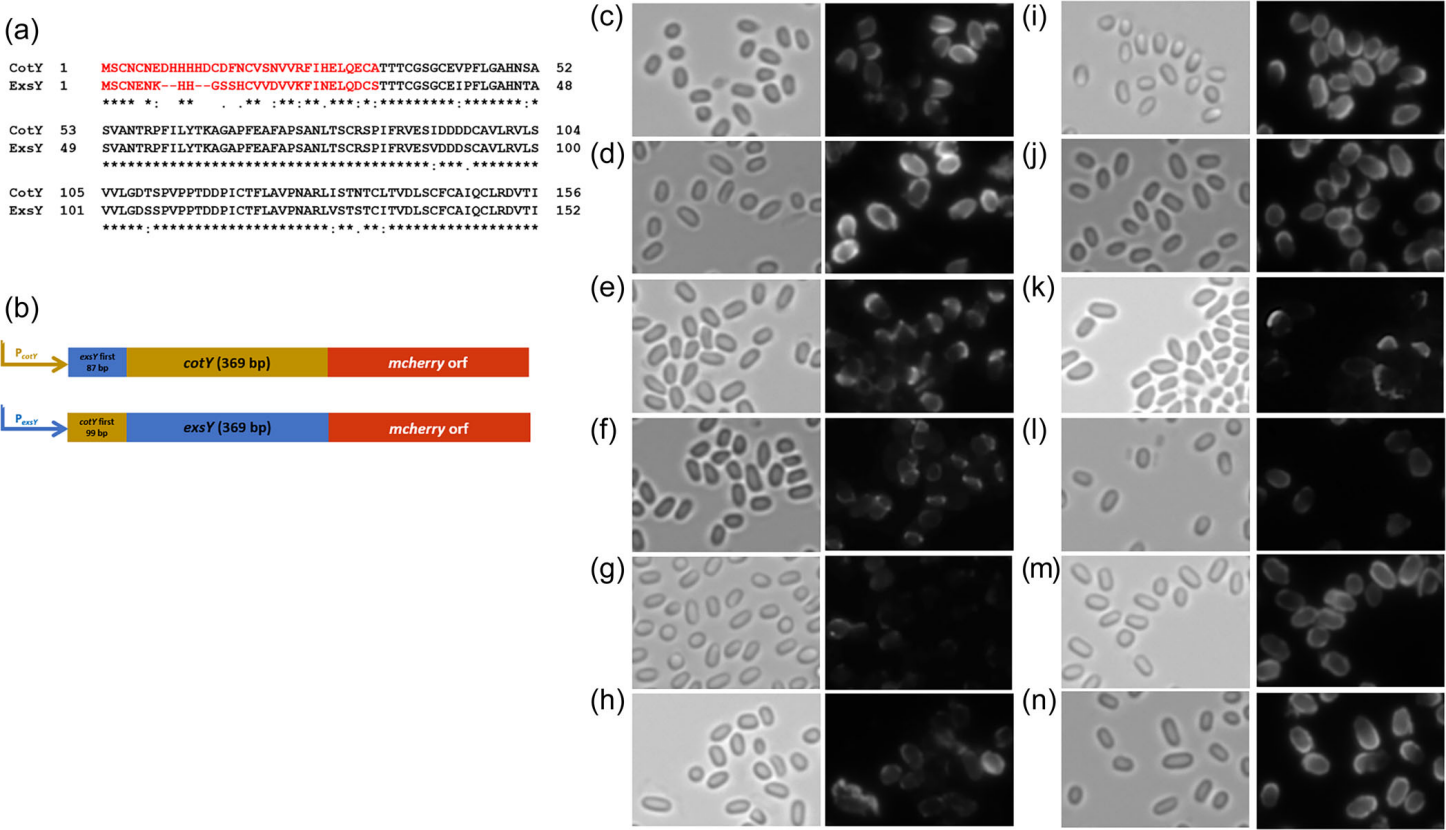
Exosporium assembly pattern of CotY and ExsY chimeric fusion proteins. (a) Sequence alignment of the *Bacillus anthracis* Sterne CotY and ExsY proteins. Identical amino acids are denoted by an asterisk and conservative substitutions are indicated by a “.” or “:”. The N‐terminal sequences are in red font and the C‐terminal sequences are in black font. (b) Diagram of the protein chimeras; (c–n) The left panel of each pair is the brightfield image and the right panel contains the corresponding fluorescence image. (c) Sterne pHPS2‐P_*exsY*_‐*exsY‐mcherry*; (d) Sterne pHPS2‐P_*exsY*_‐*cotY NT‐exsY CT‐mcherry*; (e) Δ*exsY* pHPS2‐P_*exsY*_‐*exsY‐mcherry*; (f) Δ*exsY* pHPS2‐P_*exsY*_‐*cotY NT‐exsY CT‐mcherry*; (g) *ΔcotY* pHPS2‐P_*exsY*_‐*exsY‐mcherry*; (h) *ΔcotY* pHPS2‐P_*exsY*_‐*cotY NT‐exsY CT‐mcherry*; (i) Sterne pHPS2‐P_*cotY*_‐*cotY‐mcherry*; (j) Sterne pHPS2‐P_*cotY*_‐*exsY NT‐cotY CT‐mcherry*; (k) Δ*exsY* pHPS2‐P_*cotY*_‐*cotY‐mcherry*; (l) Δ*exsY* pHPS2‐P_*cotY*_‐exs*Y NT‐cotY CT‐mcherry*; (m) *ΔcotY* pHPS2‐P_*cotY*_‐*cotY‐mcherry*; and (n) Δ*cotY* pHPS2‐P_*cotY*_‐*exsY NT‐cotY CT‐mcherry*.

The products of paralogous genes *cotY* and *exsY*, CotY and ExsY, respectively, are structural components of the basal layer of the exosporium. To examine spore localization of CotY and ExsY, a fluorescent fusion approach was undertaken. mCherry or eGFP coding sequences were fused in‐frame to the 3′ end of the open reading frames and introduced into *B. anthracis* Sterne on the pHPS2 or pMK4 shuttle plasmids. The CotY fusion protein localized primarily at one pole of the spore (Figure 1a). This was determined to be the exosporium cap region by its localization at the mother cell central spore pole (Thompson et al., 2012). Weaker fluorescence was evident around the noncap portion of the exosporium, indicating that lesser incorporation occurred in this region of the exosporium. Because of its overall weaker fluorescence, the CotY‐eGFP spores exhibit more of a cap‐only pattern, with the more minor noncap incorporation being less apparent. The distribution pattern of the ExsY fusions depended on the nature of the fluorescent reporter. ExsY‐eGFP fusions localized around the spore periphery. However, the ExsY‐mCherry fusions labeled ~75% of the spore surface with a paucity of fluorescence at one pole (a noncap region distribution pattern) (Figure 1). Spores from Sterne cells harboring compatible plasmids encoding CotY‐mCherry and ExsY‐eGFP produced spores with CotY‐mCherry present at one pole and ExsY‐eGFP predominantly at the noncap portion of the exosporium (Figure 1b). Table 1 provides a summary of the findings from the spore images shown in Figures 1, 2, 3, 4, 5.

**Table 1:** Summary of CotY and ExsY fusion protein spore localization patterns^a^

Expressed gene in spore	Host	Protein localization	Comments	Figures
P_*cotY*_ *‐cotY‐mCh*	Sterne	Bottlecap pole exosporium basal layer	Wild‐type pattern	1
P_*exsY*_ *‐exsY‐mCh*	Sterne	Noncap exosporium basal layer	Wild‐type pattern	1
P_*cotY*_ *‐cotY‐mCh*	*ΔcotY*	Entire exosporium basal layer	Loss of cap pole‐only localization	2
P_*cotY*_ *‐cotY‐mCh*	*ΔcotY ΔexsY*	Poor incorporation; likely at interspace region	Evidence that CotY‐mCherry does not functionally replace CotY to restore exosporium assembly to the double mutant	2
P_*exsY*_ *‐exsY‐mCh*	*ΔexsY*	Bottlecap pole exosporium basal layer	Evidence that ExsY‐mCherry does not functionally replace ExsY to restore noncap exosporium assembly to the *exsY* null mutant	2,3
P_*exsY*_ *‐exsY‐mCh*	*ΔcotY ΔexsY*	Poor incorporation; likely at interspace region	Evidence that ExsY‐mCherry does not functionally replace ExsY to restore bottlecap exosporium assembly to the double mutant	2
P_*exsY*_ *‐mCh‐exsY*	Sterne	Poor incorporation	Fusion of mCherry to the ExsY N‐terminus results in poor exosporium incorporation in the presence of unfused ExsY protein	4
P_*exsY*_ *‐mCh ‐exsY*	*ΔexsY*	Weak incorporation at exosporium bottlecap pole	Fusion of mCherry to the ExsY N‐terminus results in relatively weak exosporium bottlecap incorporation in the absence of unfused ExsY protein	4
P_*exsY*_‐*cotY NT‐exsY CT‐mCh*	Sterne	Entire exosporium and noncap localization patterns	Similar overall distribution pattern as the wild‐type ExsY‐mCherry	5
P_*exsY*_‐*cotY NT‐exsY CT‐mCh*	*ΔexsY*	Exosporium bottlecap pole localization	Weaker overall labeling than seen with the wild‐type ExsY‐mCh fusion protein	5
P_*exsY*_‐*cotY NT‐exsY CT‐mCh*	*ΔcotY*	Weak patchy incorporation into the exosporium basal layer	Presence of wild‐type CotY protein is needed for efficient incorporation of the chimeric fusion protein.	5
P_*cotY*_ *‐exsY* NT‐*cotY* CT‐*mCh*	Sterne	Entire exosporium basal layer	Similar to nonchimeric CotY‐mCh pattern	5
P_*cotY*_ *‐exsY* NT‐*cotY* CT‐*mCh*	*ΔexsY*	Bottlecap pole incorporation and fainter incorporation evident at noncap (exosporiumless) region of spore	Weaker incorporation at bottlecap in the absence of the unfused ExsY protein; noncap labeling likely due to interspace layer incorporation as no exosporium is present at this spore site.	5
P_*cotY*_ *‐exsY* NT‐*cotY* CT‐*mCh*	*ΔcotY*	Entire exosporium basal layer	Similar to nonchimeric CotY‐mCh pattern	5
P_*exsY*_‐*exsY NT*‐*mCh*	Sterne	Weak labeling around the spore surface	Faint relative to that of the full‐length ExsY‐mCherry fusion protein	6
P_*exsY*_‐*exsY CT*‐*mCh*	Sterne	Robust incorporation around the spore periphery	Similar to that of full‐length ExsY‐mCherry fusion protein in pattern, but reduced overall fluorescence	6
P_*exsY*_‐*exsY NT*‐*mCh*	*ΔexsY*	Faint incorporation around entire spore	Similar to that of Sterne	6
P_*exsY*_‐*exsY CT*‐*mCh*	*ΔexsY*	Predominantly bottlecap pole only labeling	Similar to full‐length ExsY‐mCh pattern	6
P_*exsY*_‐*exsY NT*‐*mCh*	*ΔcotY*	Faint incorporation around entire spore	Similar to that of the ExsY ‐ NT ‐ mCh fusion pattern	6
P_*exsY*_‐*exsY CT*‐*mCh*	*ΔcotY*	Robust incorporation around entire spore	Similar to that of Sterne	6
P_*cotY*_‐*cotY NT*‐*mCh*	Sterne	Faint incorporation around entire spore	Better incorporation levels than that of the ExsY ‐ NT ‐ mCh localization patterns	6
P_*cotY*_‐*cotY CT*‐*mCh*	Sterne	Bottlecap pole localization	More uniform bottlecap pole only than that of the full‐length CotY‐mCh fusion pattern	6
P_*cotY*_‐*cotY NT*‐*mCh*	*ΔexsY*	Faint incorporation around entire spore; stronger signal at bottlecap pole	Weaker incorporation than with the full‐length fusion protein	6
P_*cotY*_‐*cotY CT*‐*mCh*	*ΔexsY*	Bottlecap pole localization	Weaker incorporation than with the full‐length fusion protein	6
P_*cotY*_‐*cotY NT*‐*mCh*	*ΔcotY*	Faint incorporation around entire spore	Better incorporation levels than that of the ExsY ‐ NT ‐ mCh localization patterns	6
P_*cotY*_‐*cotY CT*‐*mCh*	*ΔcotY*	Faint, patchy incorporation	Poor incorporation in the absence of the wild‐type CotY protein	6
P_*cotY*_‐*exsY*‐*mCh* (or ‐*eGFP)*	Sterne	Bottlecap pole localization	The *cotY* promoter results in ExsY exhibiting the CotY spore distribution pattern	7
P_*exsY*_‐*cotY*‐*mCh* (or *eGFP)*	Sterne	Noncap exosporium basal layer localization	The *exsY* promoter results in CotY exhibiting the ExsY spore distribution pattern	7

^a^ Results from the plasmid expression experiments. Results for the promoter exchange experiments and the wild‐type fusion proteins were verified with the single copy chromosomal ectopic insertions, as shown in Figures 9 and 10.

**Figure 6:**
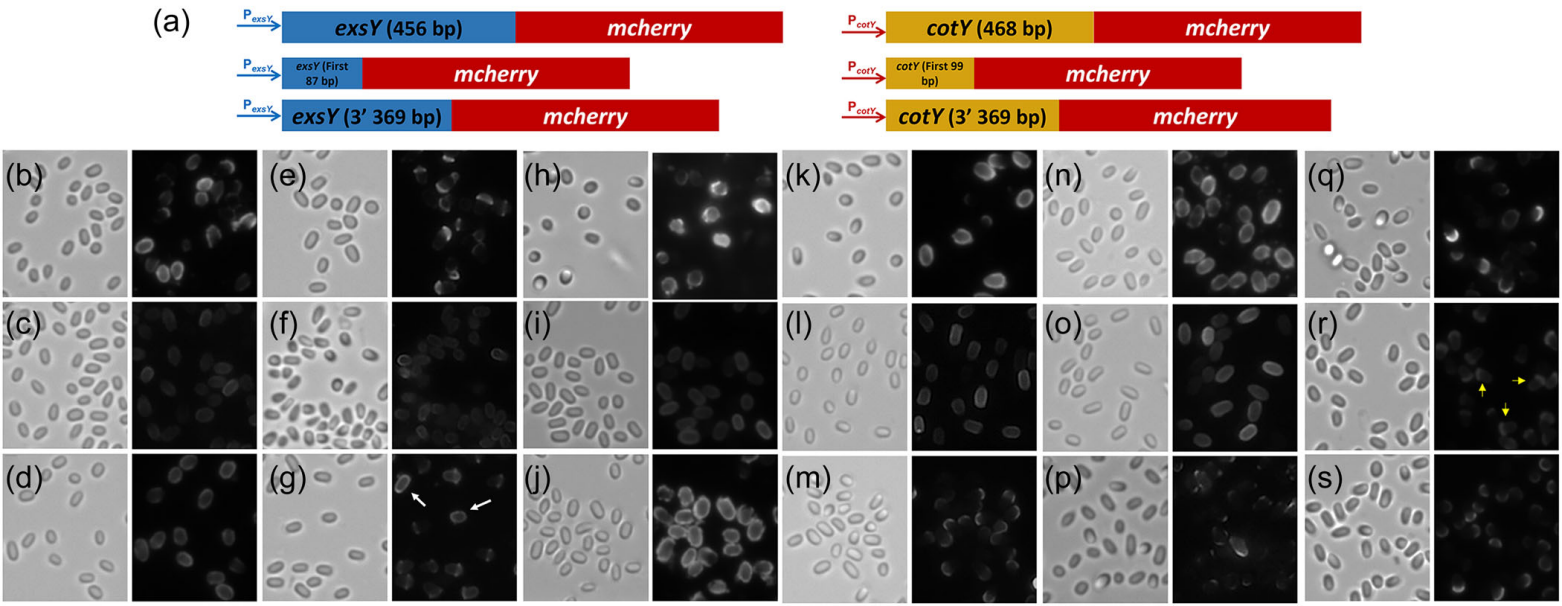
Exosporium assembly pattern of ExsY and CotY truncated fusion proteins. (a) Design of the NT and CT fusion constructs; (b–s) The left panel of each pair is the brightfield image and the right panel contains the corresponding fluorescence image. (b) Sterne pHPS2‐ P_*exsY*_‐*exsY*‐*mcherry*; (c) Sterne pHPS2‐P_*exsY*_‐*exsY NT*‐*mcherry*; (d) Sterne pHPS2‐P_*exsY*_‐*exsY CT*‐*mcherry*; (e) Δ*exsY* pHPS2‐P_*exsY*_‐*exsY‐mcherry*; (f) Δ*exsY* pHPS2‐P_*exsY*_‐*exsY NT‐mcherry*; (g) Δ*exsY* pHPS2‐P_*exsY*_‐*exsY CT‐mcherry*; (h) Δ*cotY* pHPS2‐P_*exsY*_‐*exsY‐mcherry*; (i) Δ*cotY* pHPS2‐P_*exsY*_‐*exsY NT‐mcherry*; (j) Δ*cotY* pHPS2‐P_*exsY*_‐*exsY CT‐mcherry*; (k) Sterne pHPS2‐P_*cotY*_‐*cotY*‐*mcherry*; (l) Sterne pHPS2‐P_*cotY*_‐*cotY* NT‐*mcherry*; (m) Sterne pHPS2‐P_*cotY*_‐*cotY* CT‐*mcherry*; (n) Δ*cotY* pHPS2‐P_*cotY*_‐*cotY*‐*mcherry*; (o) Δ*cotY* pHPS2‐P_*cotY*_‐*cotY* NT‐*mcherry*; and (p) Δ*cotY* pHPS2‐P_*cotY*_‐*cotY* CT‐*mcherry*; (q) Δ*exsY* pHPS2‐P_*cotY*_‐*cotY*‐*mcherry*; (r) Δ*exsY* pHPS2‐P_*cotY*_‐*cotY NT*‐*mcherry*; (s) Δ*exsY* pHPS2‐P_*cotY*_‐*cotY CT*‐*mcherry*. The white arrows in (g) indicate positions of spores where the fusion protein was positioned around the entire spore periphery, rather than at one pole. The yellow arrows in (r) point out the stronger fluorescence at the cap, but faint fluorescence is evident in the noncap portion of the exosporium.

**Figure 7:**
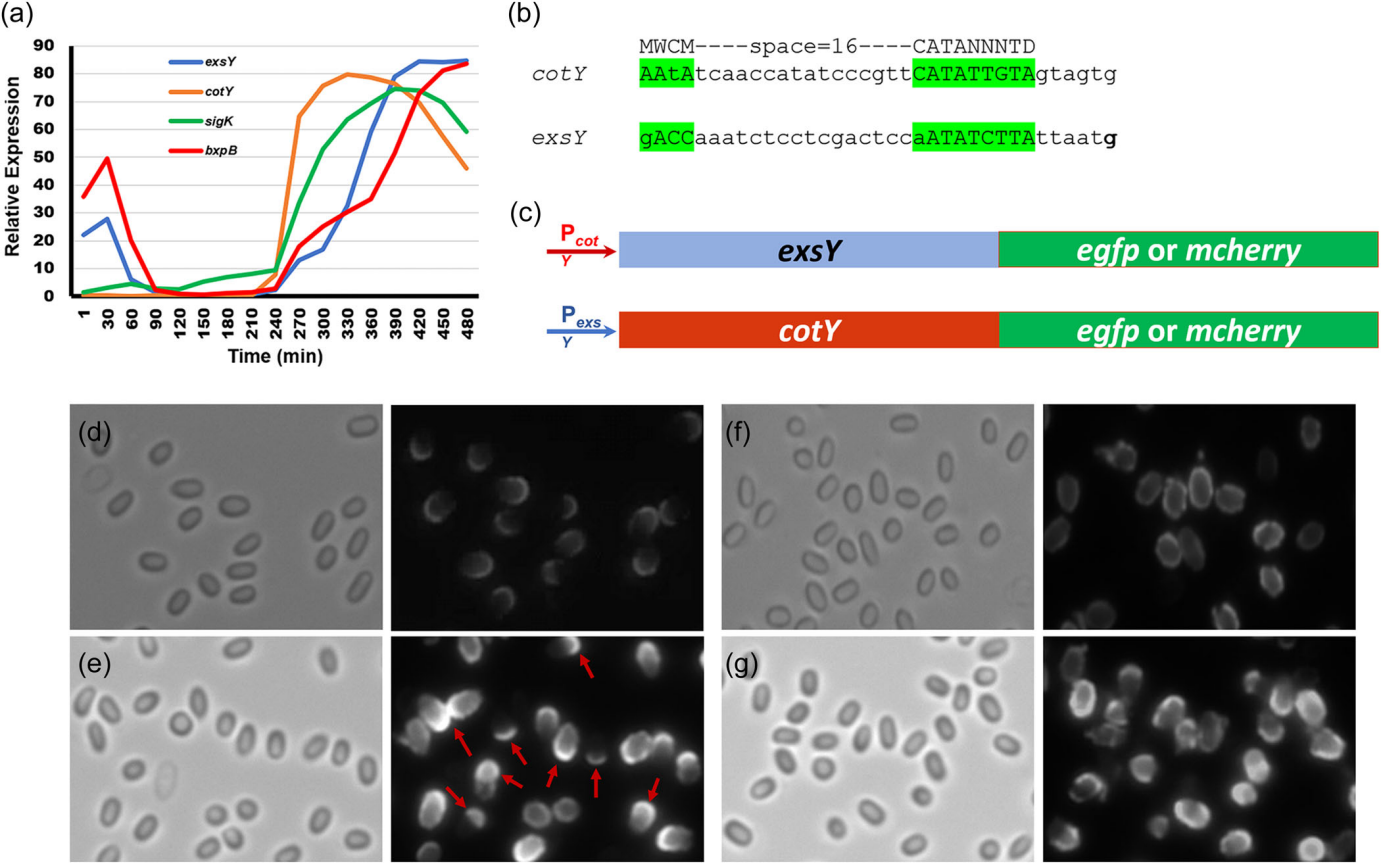
Impact of transcription timing on CotY and ExsY localization. (a) Microarray expression data from Bergman et al. (2006). The expression of the *sigK* determinant, which encodes the sigma K factor responsible for transcription of late sporulation genes in the mother cell and that of the *bxpB* exosporium basal layer determinant, are shown as sporulation time point references. (b) The sigma K promoter sequences of the *cotY* and *exsY* genes with the σ^K^ consensus sequences shown in green highlights. Matches to the consensus sequence are shown in upper case. The transcription start site defined by Peng et al. (2016) for the *exsY* gene is shown in bold font. (c) Promoters from *cotY* and *exsY* were exchanged and the mCherry fusion proteins were expressed off of the pHPS2 plasmid in *Bacillus anthracis* hosts. Spore localization patterns were examined. The left panel of each pair is the brightfield image and the right panel contains the corresponding fluorescence image. (d) Sterne pHPS2‐P_*cotY*_‐*exsY*‐*egfp*; (e) Sterne pHPS2‐P_*cotY*_‐*exsY*‐*mcherry*; (f) Sterne pHPS2‐ P_*exsY*_‐*cotY*‐*egfp*; and (g) Sterne pHPS2‐P_*exsY*_‐*cotY*‐*mcherry*.

Figure 1 shows the predominant localization of CotY and ExsY fusion proteins in Sterne spores, which also contain unfused copies of the CotY and ExsY proteins. We next examined the localization patterns of the fusion proteins in mutants deleted for *cotY*, *exsY*, or both (Figure 2). The CotY‐mCherry fusion protein was distributed uniformly around the Δ*cotY* spores (Figure 2a). The predominance of labeling at the cap pole seen with the Sterne host is not apparent in the mutant spores lacking unfused CotY protein. In this mutant background, the exosporium cap is not produced early in sporulation, and as is shown below, the CotY fusion protein cannot complement the *cotY* null mutant. With the *cotY exsY* double mutant spores, the incorporation of the CotY fusion protein is very weak but evident around the entire periphery of the spore (Figure 2b). The phenotype of the double mutant is a lack of an exosporium (Durand‐Heredia et al., 2022; Johnson et al., 2006). If the *cotY‐mcherry* determinant was able to complement the *cotY* deletion, then the majority of spores would be phenotypically ExsY‐negative and CotY‐positive and only the bottlecap portion of the exosporium would form and incorporate the CotY‐mCherry fusion protein. The lack of cap‐only labeling suggests the CotY‐mCherry is not functional and thus fails to complement. Nonspecific adherence to the exosporium‐less spores cannot be ruled out as an explanation for this weak labeling of the spores. However, nonspecific adsorption would be expected to give a less uniform and more mottled fluorescence appearance.

### 3.2 The ExsY‐mCherry fusion protein is not fully functional in basal layer assembly

Spores from an *exsY* deletion mutant produce only the exosporium cap region (Boydston et al., 2006; Johnson et al., 2006). Expression of the ExsY‐mCherry fusion protein in this genetic background resulted in the presence of fluorescence in the exosporium cap, but not in the noncap portion of the exosporium (Figure 2C). When expressed in the *cotY exsY* double mutant strain, ExsY‐mCherry exhibited weak and patchy incorporation over the spore surface (Figure 2D). The results of ExsY‐mCherry expression in the single and double mutant strains are consistent with the fusion lacking full functionality, and thus the fusion gene fails to complement the *exsY* null mutation, thus retaining the cap‐only fluorescence. The faint incorporation in the double mutant spores could be due to the nonspecific binding of the fusion protein to the exosporium‐less spores. However, because the ExsY‐mCherry protein was not detected in the noncap portion of the Δ*exsY* spores, it suggests this fusion protein does not nonspecifically adhere to areas of the spore lacking the exosporium. Thus, nonspecific adherence to the surface of the Δ*cotY* Δ*exsY* spores seems to be an unlikely explanation for the weak fluorescence observed. As further evidence in support of the *exsY*‐*mcherry* determinant not complementing the *exsY* deletion mutation, BclA was used as an indicator for the presence of the exosporium. Spores were prepared from the *exsY* null mutant, and the null mutant bearing either the pHPS2 plasmid encoding the ExsY protein or the ExsY‐mCherry fusion protein. Spores were reacted with anti‐BclA antiserum and the anti‐rabbit IgAlexa Fluor 568 conjugate. The results are shown in Figure 3. The mutant lacking ExsY produces only the cap portion of the exosporium and is complemented by the plasmid‐borne *exsY* determinant, as evident from the presence of the BclA nap protein around the spore. The fusion protein‐containing spores, however, lack the noncap portion of the exosporium.

### 3.3 Fluorescent tags at the N‐terminus of CotY and ExsY are poorly incorporated onto spores

When the eGFP or mCherry tags were added to the N‐terminus of CotY or ExsY in the Sterne host, fluorescence was barely above background levels. This is shown for mCherry‐ExsY in Figure 4. When the mCherry‐ExsY fusion protein was expressed in the *ΔexsY* host, the spores, weak fluorescence was evident in the margin of the bottlecap (Figure 4d–f). There was no detectable fluorescence in the dome of the cap structure, suggesting that the mCherry‐ExsY fusion did not compete with CotY for assembly in the cap and could only be added at the margin of the completed cap. In panels (g, h), a sporulating cell is shown with released spores, showing that the mCherry‐ExsY protein is present at the mother cell‐central pole (bottlecap) of the spore, but the released spores exhibit much less fluorescence, suggesting that the fusion protein is not stably assembled into the basal layer of the cap. It appears that the presence of the unfused ExsY protein outcompetes the mCherry‐ExsY fusion protein for incorporation into the developing exosporium basal layer. Cells from sporulating cultures of the same age expressing mCherry‐ExsY (panel j) and ExsY‐mCherry (panel k) are shown. The N‐terminus fluorescent fusion proteins remain predominantly in the cytoplasm of the cells, whereas the C‐terminus fusion proteins have been incorporated into the exosporium, leaving little fluorescent signal in the cytoplasm. This supports that the fusions with the mCherry at the ExsY N‐terminus are defective for assembly into the exosporium when unfused ExsY is present. Also shown is evidence that the mCherry protein alone does not adsorb onto spores, even when expressed at high levels in sporulating cells off of the *bclA* promoter (panels l–n).

The mCherry‐CotY fusion protein likewise gave barely detectable fluorescence when expressed in Sterne sporulating cells. Because of the poor incorporation of the N‐terminus fusions, the localization studies shown below utilized fluorescent fusions to the C‐terminus of the CotY and ExsY proteins.

### 3.4 The N‐terminal sequences of CotY and ExsY do not fully account for the differential localization patterns of the two proteins

The CotY and ExsY proteins are very similar in amino acid composition, with an overall amino acid sequence identity of 85.9% (134 identical residues out of 156) and with 146 out of 156 residues being similar (93.6%, Figure 5a). The N‐terminus sequences exhibit the greatest heterogeneity. The first 33 residues of CotY, corresponding to residues 1–29 of ExsY) are only 51.5% identical and CotY has four consecutive histidine residues, whereas only two histidines are present at this location in ExsY. The remaining 123 residue portion of the two proteins are 93.5% identical and 99% similar. Although there are no crystal structures for the two proteins, AlphaFold predictions (https://alphafold.ebi.ac.uk) are quite similar except for the N‐terminal sequences (but which are low‐confidence predictions). We investigated if this N‐terminal sequence heterogeneity was important in the differential positioning of the two proteins in the exosporium basal layer.

We engineered two chimeric genes by fusing the N‐terminal coding sequences of the *exsY* ORF to the C‐terminal coding sequences of the *cotY* ORF and the N‐terminal coding sequences of the *cotY* ORF to the C‐terminal coding sequences of the *exsY* ORF. These chimeric genes were introduced on plasmids into the Sterne strain and spores prepared. The expression of the chimeric fusions was driven by the promoter that corresponds to the chimeric gene carboxy‐terminus coding sequence because the idea was to determine if the chimeric N‐terminal sequences could dictate the locational fate of the resultant protein. The fluorescence patterns exhibited by the chimeric protein expressing spores are presented in Figure 5. Wild‐type ExsY localizes predominantly at the noncap region in Sterne spores, giving a “U” shape appearance encompassing approximately 75% of the spore surface (Figure 5c). The CotY NT‐ExsY CT chimeric fusion protein gave the same overall distribution pattern as the wild‐type ExsY‐mCherry protein (Figure 5c,d). Although the incorporation was strong with the chimeric protein, its incorporation gave a more mottled appearance than with the wild‐type fusion protein and the labeling of the cap region was more pronounced. In the *exsY* deletion mutant host, the substitution of the CotY N‐terminus for the ExsY NT sequence resulted in reduced fluorescence in the exosporium cap (compare panels e and f of Figure 5). In the *cotY* null background, the ExsY‐mCherry protein was poorly incorporated into the spores and displayed a mottled appearance (Figure 5g). This result identifies a defect in the fusion protein. *ΔcotY* spores produce an intact ExsY‐containing exosporium. Thus, unfused ExsY can incorporate into the exosporium, whereas ExsY‐mCherry is less able to do so. Substitution with the CotY NT sequence improved fusion protein incorporation levels in the *cotY* null mutant spores (Figure 5h), but they were still weak compared to levels observed in the Sterne strain background.

Wild‐type CotY‐mCherry was distributed around the spore with enrichment at the cap pole or, in a subset of spores, predominantly at the cap pole in the Sterne strain spores. Replacement of the N‐terminus sequence with that of ExsY had no appreciable effect on incorporation levels or distribution in this wild‐type strain (Figure 5i,j). With the *ΔexsY* mutant host, there was a substantial difference in the spore localization pattern of CotY‐mCherry versus ExsY NT‐CotY CT‐mCherry (Figure 5k,l). The wild‐type CotY‐mCherry fusion protein exhibited fluorescence only at the exosporium cap (Figure 5k). The presence of the ExsY NT sequence resulted in reduced fluorescence at the cap, but enhanced fluorescence, although still weak, into the noncap portion of the spore. Stronger labeling in the noncap portion of the spore is surprising given the lack of an exosporium at this location in the *exsY* null spores. It suggests that the ExsY NT sequence may be interacting with a component of the interspace region of the spore.

With *cotY* null spores, there was no appreciable difference between the localization patterns of the CotY‐mCherry and the ExsY NT‐CotY CT‐mCherry proteins (Figure 5m,n). The CotY fusion proteins do not appear to have the difficulty of incorporation into the *cotY* null spores as seen with the ExsY fusion protein.

The N‐terminus sequences of the CotY and ExsY proteins were not sufficient to account for the different localization patterns of the two proteins. We continued investigating possible roles for the N‐terminal sequences of CotY and ExsY, but this time using a truncated fusion protein approach. These constructs are illustrated in Figure 6a. The full‐length and truncated genes were fused in‐frame with the mCherry ORF. Spores were prepared from the Sterne, *ΔcotY*, and *ΔexsY* strains expressing the fusion constructs. Sterne spores that expressed the ExsY NT fusion displayed weak overall labeling around the spore surface (Figure 6c), while ExsY CT‐mCherry fusions exhibited more robust incorporation around the spore periphery (Figure 6d). ExsY and ExsY CT fusions give similar patterns of localization, but the full‐length fusion gives a stronger overall fluorescence. Full‐length ExsY‐mCherry was found only at the cap in *exsY* null spores, indicating that the fusion gene fails to complement the deletion mutation and produces an intact exosporium (Figure 6e). The signal of ExsY NT fusions in *exsY* null spores gives the same weak spore periphery pattern as with Sterne, which includes fluorescence in the noncap portion of the spore that lacks the exosporium layer (Figure 6f). The ExsY CT fusion in the *ΔexsY* background predominantly labeled only the cap (Figure 6g). However, a small subset of spores exhibited fluorescence over the entire spore surface (denoted by arrows in Figure 6g). The ExsY CT fusion protein was less efficiently incorporated into the cap basal layer than full‐length ExsY‐mCherry. With the *cotY* null spores, the ExsY‐CT fusion protein more strongly incorporated into the exosporium basal layer encompassing the entire spore than did the ExsY full‐length fusion protein (compare Figure 6h,j), whereas the NT fusion gave the faint incorporation around the spore (Figure 6i), similar to that seen with Sterne spores. The full‐length protein's incorporation was weaker and more mottled in appearance (Figure 6h) than that of the ExsY CT fusion (panel j), and the intensity of the fluorescence among the former spores was weaker overall and more variable.

The full‐length CotY‐mCherry fusion localized heterogeneously around the Sterne spores, with complete, partial, and pole‐only patterns evident (Figure 6k). Expression of the CotY NT fusion in Sterne spores was less robust, but more uniformly encompassed the entire surface of the spores (compare Figures 6k,l). The fluorescent signal from the CotY NT fusion was stronger than that seen with the ExsY NT fusion (compare panels g, f, and i with l, o, and r). This may result from the degenerate changes in the amino acid sequence (including deletion of two histidine residues) that may weaken the ExsY NT interaction with the interspace binding partner(s).

The CotY CT fusion in the Sterne background presented as a cap‐only pattern with the overall incorporation being relatively weak (Figure 6m). This suggests reduced interactions with CotY in the cap and the lack of fluorescence beyond the cap suggests a loss of interaction with ExsY as is often seen with pHPS2‐based expression of CotY‐mCherry. In the *cotY* null background, the distribution patterns of the full‐length CotY‐mCherry and CotY NT‐mCherry fusion proteins remained similar to that found in the Sterne hosts (Figure 6n,o). However, the incorporation of the CotY CT‐mCherry fusion protein was dependent on the presence of the wild‐type CotY protein. With the *cotY* null spores, the CotY CT‐mCherry fusion protein did not localize to the cap but appeared in the spores as small patches located heterogeneously at polar or subpolar sites (compare Figure 6m,p). The presence of the CotY N‐terminus sequence is important for localization to the exosporium cap in the absence of the wild‐type CotY protein. With the *exsY* null spores, the full‐length CotY‐mCherry and CotY CT‐mCherry proteins localize at the cap. The CotY NT‐mCherry protein appears in the cap, as well as fainter incorporation in the noncap (exosporium‐less) portion of the spore.

The results support a role for the N‐terminus sequences of both CotY and ExsY contributing to the assembly of these basal layer proteins into the exosporium. The N‐terminus sequences of both proteins direct incorporation around the spore periphery, even in the absence of the noncap basal layer with the *exsY* null mutant spores. The presence of the wild‐type CotY or ExsY proteins affects the level of NT fusion incorporation, but not the pattern. This suggests that short NT sequences can partner with a protein or proteins, likely in the interspace. The CotY CT sequence directs the fusion protein predominantly to the cap spore pole, but assembly at this site is dependent on the presence of the full‐length protein.

### 3.5 The impact of the timing of *cotY* and *exsY* transcription on their patterns of incorporation into the *B. anthracis* exosporium basal layer

Because the impact of the N‐terminus sequences failed to fully explain the differences in basal layer localization patterns of CotY and ExsY, we next chose to examine if there was an impact of timing of expression of the two genes on the spore localization outcome. Bergman et al. (2006) conducted a microarray analysis of transcription patterns with *B. anthracis*, including during the sporulation stages. Their results for expression of *cotY* and *exsY* (along with the mother cell's specific late sporulation sigma factor σ^K^) are shown in Figure 7a. Both *cotY* and *exsY* are transcribed by RNA polymerase containing the σ^K^ sigma factor (Peng et al., 2016). Transcription of *cotY* precedes that of *exsY* by about 20 min and messenger RNA (mRNA) levels begin to decline after approximately 80 min. The expression of *exsY* peaks at a time when *cotY* expression is rapidly declining and levels of *exsY* mRNA remain elevated throughout the late stages of spore assembly. To determine if the timing and/or levels of expression impact the patterns of assembly of the CotY and ExsY proteins, we exchanged the promoter elements (the promoter sequences and ribosome binding site sequences) such that the *exsY* fusion determinant was expressed from the *cotY* promoter and the *cotY* fusion determinant was expressed using the *exsY* promoter. The results from the fluorescent analysis of these spores are shown in Figure 7.

Expression of *exsY‐egfp* or *exsY‐mcherry* from the *cotY* promoter in Sterne cells resulted in spores with fluorescence patterns identical to the CotY localization pattern (Figure 7c,d). Fluorescence was predominantly localized to the cap, with weaker fluorescence in the noncap region of the spore. Conversely, expression of *cotY‐egfp* or *cotY‐mcherry* from the *exsY* promoter in Sterne cells resulted in spores with fluorescence patterns identical to the ExsY localization pattern (Figure 7e,f). The fluorescence was observed around the periphery of the spore, with a subset of the spores bearing a noncap expression pattern with reduced incorporation of the fusion protein in the cap pole basal layer. The fluorescence formed a mottled appearance, again similar to what we observed with ExsY‐mCherry expressed off of its native promoter on the pHPS2 plasmid. The results indicate that timing of expression plays a critical role in the assembly pattern of CotY and ExsY in the developing spore exosporium basal layer.

### 3.6 The *B. anthracis exsY* promoter region

**Figure 8:**
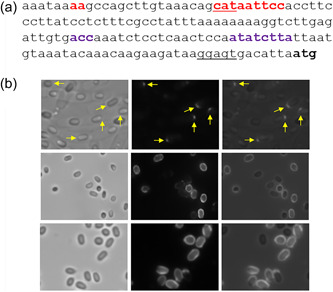
(a) The nucleotide sequence of the *exsY* (bas1141) promoter region of the Sterne strain of *Bacillus anthracis*. The putative upstream σ^K^ promoter sequence is in red font and the downstream promoter is in purple font. The *Bacillus* σ^K^ consensus sequence is MWCM‐space=16‐CATANNNTD, where D is A, G, or T; N is A, C, G, or T; M is A or C; and W is A or T. The start codon of the divergently transcribed *bas1142* determinant, which lies within the putative upstream *exsY* promoter element is underlined. The *exsY* ribosome binding site is underlined and the start codon is indicated in bold font. (b) Brightfield, fluorescent, and merged images of spores from strains expressing ExsY‐mCherry from only the upstream promoter (top row), only the downstream promoter (middle row), and from the intact promoter region (bottom row). The top row is a mixture of sporulating cells and spores, showing that the fluorescence was observed at one pole of the developing spores and that no fluorescence was evident in the released spores. Yellow arrows denote locations where the cap pole fluorescence was observed in spore‐bearing mother cells.

The *B. anthracis exsY* determinant is transcribed by RNA polymerase bearing the σ^K^ sigma factor. There are two putative σ^K^ promoter sequences upstream of the *exsY* ORF. The better match to the consensus sigma‐K promoter is the downstream one. The upstream putative element, which overlaps the start of the *bas1142* ORF, has a poor match at the “−35” element (Figure 8). The start site of transcription for the *exsY* gene of *Bacillus thuringiensis* has been mapped to the “G” nucleotide immediately distal to the downstream σ^K^ promoter element shown in Figure 8a (Peng et al., 2016). The Sterne sequence is identical to that of *B. thuringiensis* except for an additional “A” nucleotide in the stretch of eight consecutive A bases, in the sequence between the putative upstream and downstream promoter elements. To determine if either or both promoters are functional in *B. anthracis*, we deleted the upstream or the downstream promoter sequences and introduced plasmids bearing *exsY‐mcherry* with the truncated promoter elements into the Sterne strain. Sporulating cells and spores were examined for fluorescence and compared to expression from a plasmid bearing the intact promoter region sequence. The downstream promoter element drove production of the fusion protein in a fashion comparable to that of the intact promoter region. The putative upstream element, however, was found to function, albeit weakly. With the upstream promoter, fluorescence was observed that localized to a discreet point on the bottlecap pole of the developing spore (Figure 8, top row). This fluorescence was only observed in sporulating cells, no fluorescence was detected in released mature spores. The results suggest that the upstream putative promoter element may be weakly functional. The fusion protein localizes to the exosporium cap site, but the fluorescence was below the limit of detection in mature spores.

### 3.7 *exsY* and *cotY* single‐copy expression patterns

**Figure 9:**
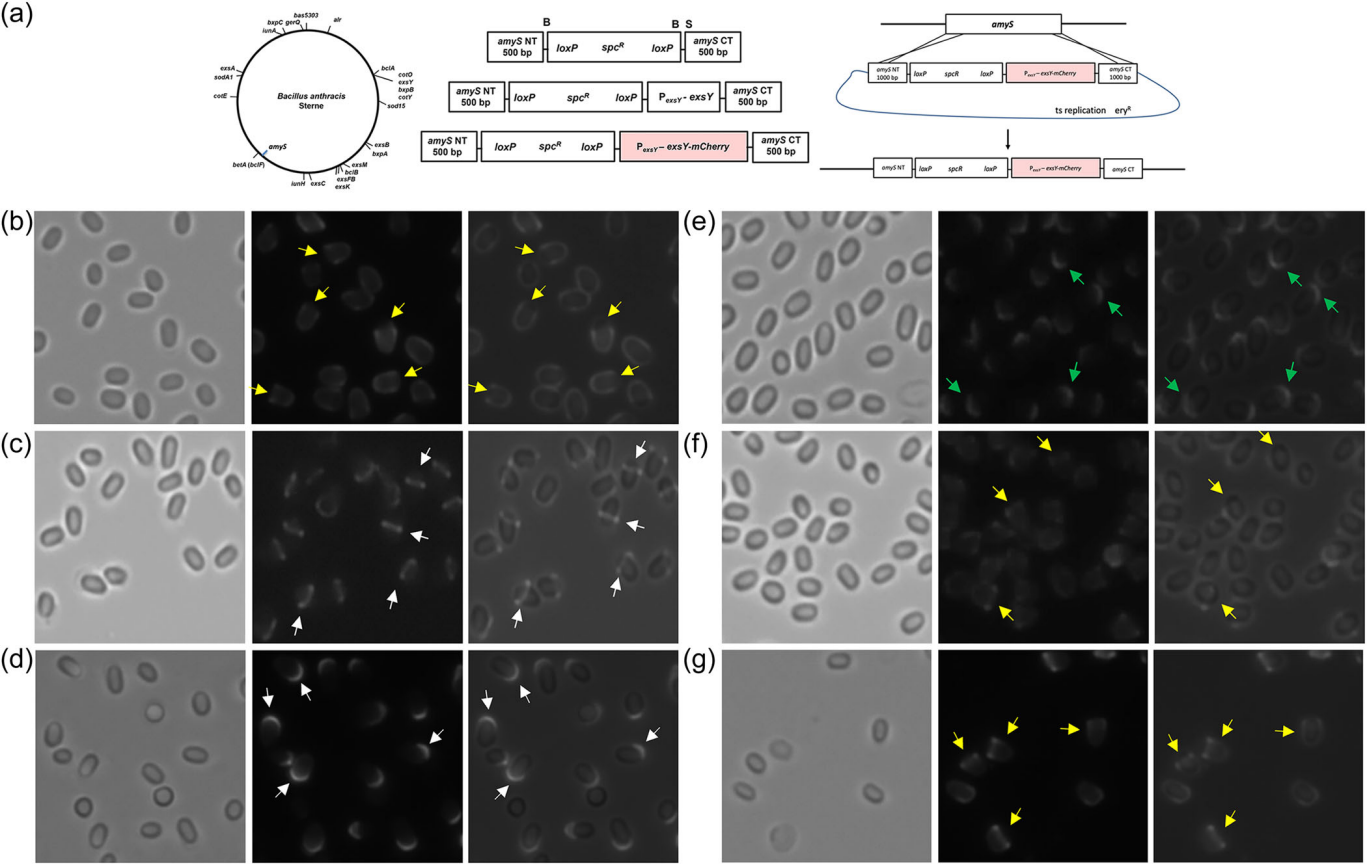
*amyS* single copy complementation system. Expression patterns of single copy chromosomal integrations of *exsY‐mcherry* and *cotY‐mcherry*. (a) Left: genome map of *Bacillus anthracis* with select exosporium determinants and *amyS* indicated; Center: Diagram of the single copy complementation system cassette (top), the *exsY* gene inserted cassette (middle), and the *exsY‐mcherry* fusion gene inserted cassette (bottom); “B” and “S” indicate *Bam*HI and *Sal*I restriction sites; diagrams are not to scale. (b–g) Represent brightfield (left), fluorescent (middle), and merged (right) images of spores. (b) Sterne Δ*amyS::spc* P_*exsY*_ *‐exsY‐mcherry*; (c) Δ*exsY* Δ*amyS::spc* P_*exsY*_ *‐exsY‐mcherry*; (d) Sterne Δ*amyS::spc* P_*cotY*_ *‐cotY‐egfp*; (e) Sterne Δ*amyS::spc* P_*cotY*_ *‐exsY‐mcherry*; (f) Sterne Δ*amyS::spc* P_*exsY*_ *‐cotY‐mcherry*; (g) Sterne Δ*amyS::spc* P_*exsY*_ *‐cotY NT‐exsY CT‐mcherry*. White arrows denote examples of spores with fluorescence primarily at the cap‐noncap boundary; yellow arrows for noncap fluorescence; and green arrows for fluorescence at the cap pole.

Given that the results of the promoter exchange experiment indicated that the timing or level of transcription impacted CotY and ExsY fusion protein localization, concerns over the use of plasmid expression systems not accurately reflecting localization patterns exist. Higher gene dosage from the shuttle plasmids may impact the timing, or levels of transcription, of these exosporium determinants. To overcome this limitation, we inserted the exosporium gene of interest in a single copy at an ectopic site (Figure 9a). The monocistronic *amyS* (*bas2931*) locus encodes an alpha‐amylase and is flanked by oppositely oriented genes. Insertion at this locus, therefore, should not significantly impact expression pattern of the flanking genes. The *cotY* or *exsY* genes (preceded by their own or the exchanged promoter) were cloned into a cassette containing 500 bp sequences of the beginning and end of the *amyS* ORF. Internal to the *amyS* sequences were a spectinomycin resistance determinant flanked by *loxP* sequences (for removal of the marker if needed), and a *Sal*I cloning site for insertion of *SalI* or *Xho*I DNA fragments. Allele replacement mutagenesis results in the chromosomal insertion of the gene of interest (Figure 9a).

Expression of ExsY‐mCherry from single‐copy integration was tested in Sterne, *ΔexsY*, and *ΔcotY* spores. The fluorescence, although weaker than with plasmid‐expressed fusions, was evident in >90% of the spores. With the plasmid‐based expression of the CotY‐ and ExsY‐fusions, the fluorescent patterns were consistent, but there was always a subset of spores that lacked fluorescence. Sterne bearing the chromosome integrated *exsY‐mcherry* spores exhibited a distinct noncap labeling pattern, with one spore pole devoid of fluorescence (Figure 9b). In contrast, with the *exsY* null spores, the fusion protein appeared as a ring at the boundary between the cap and the noncap regions (Figure 9c). This indicates that the ExsY‐mCherry‐encoding determinant is unable to complement the *exsY* deletion mutation and restore the intact exosporium. The narrow ring of incorporation suggests that ExsY‐mCherry can associate with the bottlecap structure at the mother cell‐central pole of the spore, but additional fusion proteins cannot be added to extend the exosporium basal layer sheet. With Sterne, the incorporation of unfused ExsY provides sites for additional incorporation of the fusion protein, and hence the noncap‐specific labeling pattern.

Single‐copy expression of the CotY‐eGFP fusion protein in the Sterne parent strain resulted in spores that showed fluorescence at the cap pole, as was observed with the plasmid‐based expression (Figure 9d). We also tested if the *exsY* and *cotY* promoter elements impacted protein localization patterns when the fusion genes were present in a single copy (compared to the plasmid results shown in Figure 7). When the single copy *exsY‐mcherry* determinant was expressed off of the *cotY* promoter, fluorescence was evident at the cap pole of the spore, rather than the noncap region seen with native promoter expression (Figure 9e). When the single‐copy *cotY‐mcherry* determinant was expressed using the *exsY* promoter, the ExsY type noncap labeling pattern of the spore was evident (Figure 9f). These results validate the findings obtained with the plasmid expression studies and further suggest that it is the timing of transcription, not overexpression per se, that drives the assembly patterns of CotY and ExsY. We also examined a CotY‐ExsY chimera construct inserted at the *amyS* locus in the Sterne host strain. The *cotY NT*‐*exsY CT* chimera expressed from the *exsY* promoter yielded spores that displayed noncap fluorescence, with a stronger signal along the cap‐noncap margin (Figure 9g). This differed from the same fusion expressed off the pHPS2 plasmid, which exhibited fluorescence around the entire spore periphery, perhaps owing to the effects of overexpression from the multicopy plasmid. The enhanced fluorescence at the cap‐noncap margin was more evident than with spores from the nonchimeric ExsY fusion‐producing strain (Figure 9b).

**Figure 10:**
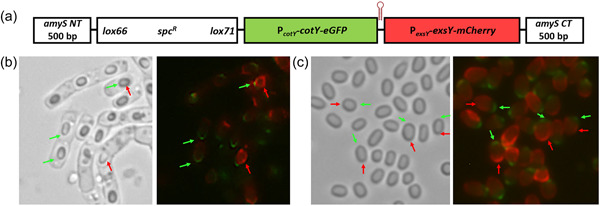
Sterne *cotY‐egfp exsY‐mcherry* single copy integration in tandem. (a) Schematic of the tandem single‐copy integration cassette bearing *cotY‐egfp* and *exsY‐mcherry*. A rho‐independent transcriptional terminator (from the *cotY‐bxpB* intergenic region) was inserted between the two fusion genes to prevent read‐through of *exsY‐mcherry* from the *cotY* promoter. (b) Sterne sporulating cells showing the fusion proteins localized primarily to the cap (CotY‐eGFP) and noncap (ExsY‐mCherry), respectively; (c) Mature spores exhibiting exosporium fluorescence. Green arrows (CotY‐eGFP); red arrows (ExsY‐mCherry).

Next, using the single‐copy integration approach we introduced, in the same cassette, consecutive fusion orfs, *cotY‐egfp* followed by *exsY‐mcherry*, each under the control of their native promoters. A transcription terminator stem‐loop sequence was inserted between the two genes to minimize bleed‐through transcription from the *cotY‐egfp* determinant. The integration cassette that was recombined into the Sterne genome is shown in Figure 10a. Expressing these fusion proteins from compatible multicopy plasmids in double‐transformed cells produced dual‐labeled spores but in only a subset of the spore population (Figure 1). Single‐labeled spores expressing one of the fusion proteins and poorly or unlabeled spores were also observed (Figure 1c). With the single copy tandem insert, both fusion proteins were expressed in the sporulating cells and appeared on the mature spores (Figure 10b,c). CotY‐eGFP localized at the cap pole while ExsY‐mCherry was found at the noncap portion of the exosporium.

### 3.8 CotY and ExsY protein interactions in a bacterial two‐hybrid system

**Figure 11:**
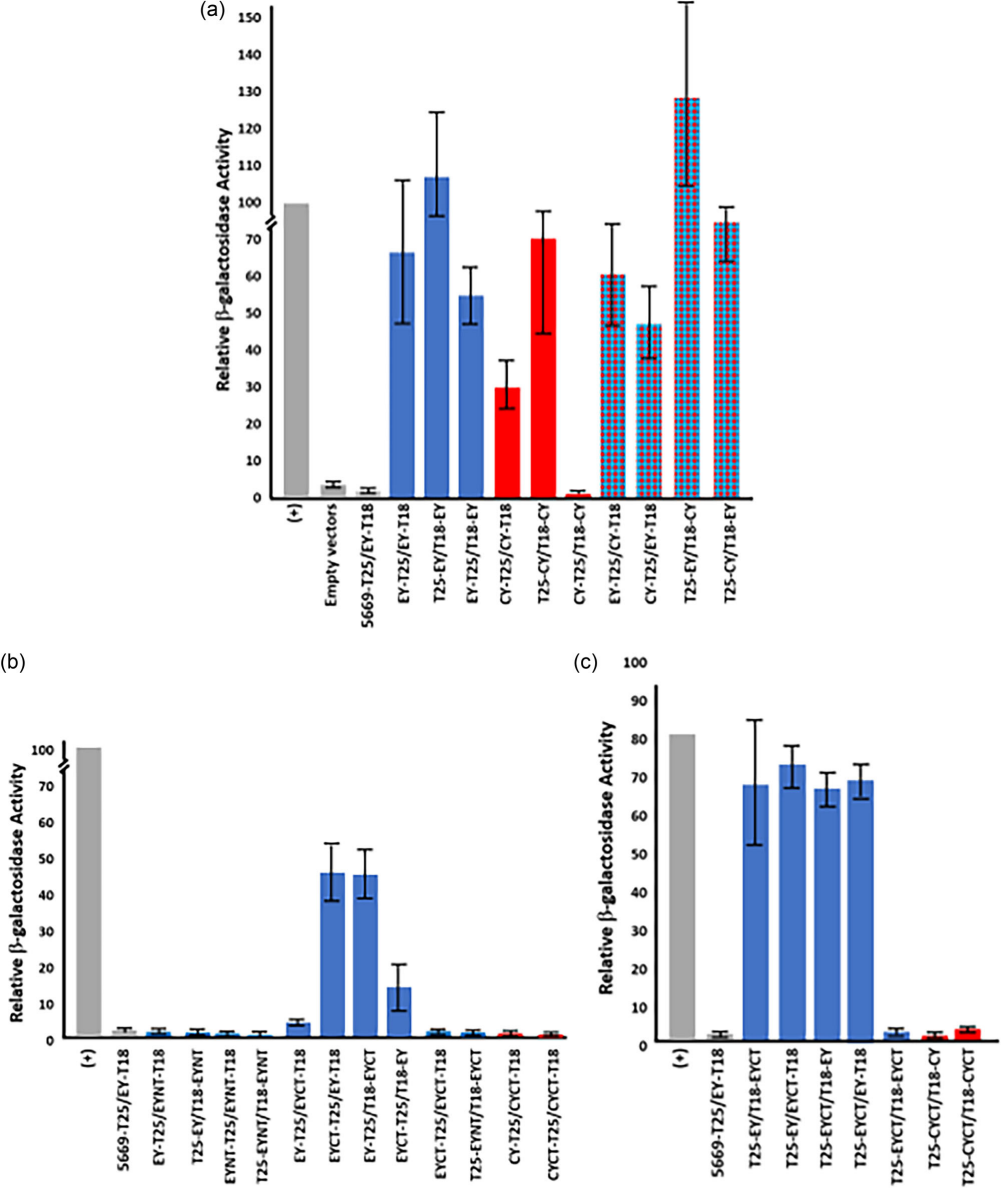
Bacterial two‐hybrid results for ExsY (blue bars), CotY (red bars), and ExsY/CotY pairs (red and blue checkerboard bars). Positive and negative controls are shown as gray bars. (A) Results with the full‐length proteins. Results with the truncated proteins are presented in (b) for the C‐terminus AC fusions and in (c) for the N‐terminus AC fusions. Values are expressed as the percentage of activity of the zip/zip positive control. Values are representative of at least three independent assays and the mean and standard deviation are shown.

To identify potential interactions between the CotY and ExsY proteins and their truncated derivatives, we used a bacterial two‐hybrid system based on adenylate cyclase from *Bordetella pertussis*. Four constructs were prepared for each examined gene with the T18 or T25 domain of adenylate cyclase fused to the C‐ or N‐terminus of every protein analyzed. Systematic screening of direct contacts was subsequently performed after transformation of all possible combinations of bait/prey plasmid pairs into the *E. coli* BTH101 strain. Protein interactions were assessed through the expression of a reporter β‐galactosidase gene. The results of these studies are shown in Figure 11. Values are expressed as a percentage of the positive control pair (T25‐Zip/T18‐Zip). Negative controls included paired empty vectors and the ExsY‐T18 protein with the AW20_5669 protein encoded on the pXO1 plasmid fused to T25. This protein is a vegetative cell‐expressed putative nucleotide sugar dehydrogenase subfamily protein that is similar in size (151 aa) to the ExsY and CotY proteins and all three are acidic proteins. The *B. cereus* ExsY and CotY His‐tagged proteins were reported to self‐assemble into sheets in the cytoplasm of the *E. coli* hosts (Terry et al., 2017). We found that the *B. anthracis* ExsY protein could productively pair with itself, regardless of whether the adenylate cyclase domain (AC) was fused to the N‐terminus or the C‐terminus of the protein (Figure 11a). The pairing was also observed with one ExsY partner having the AC at the N‐terminus and the other having the AC at the C‐terminus. The N‐terminus AC fusion pair gave positive values comparable to those of the C‐terminus fusions, suggesting that the reduced spore fluorescence observed with the N‐terminal mCherry fusions may not have been due to a defect in the pairing of the N‐terminal fusion proteins.

The CotY fusion proteins were also able to interact, with the N‐terminus AC fusions yielding substantially higher β‐galactosidase activities. Pairing an N‐terminus AC fusion with a C‐terminus AC CotY fusion gave no evidence of protein‐protein interaction, unlike the result with the ExsY pairs.

Coexpression of the CotY and ExsY fusions resulted in positive protein–protein interactions with the N‐terminus fusion pairs, the C‐terminus fusion pairs, and when an N‐terminus fusion was paired with a C‐terminus fusion (Figure 11a, checkerboard bars).

We examined potential interactions with the truncated derivatives of ExsY and CotY (Figure 11b,c). The 29‐residue ExsY N‐terminus sequence gave no evidence of partnering with full‐length ExsY, the C‐terminus protein, or with itself. The 123 residue C‐terminus ExsY sequence was found to be capable of partnering with the ExsY full‐length protein, although the magnitude of the β‐galactosidase response was variable with the AC domains at the C‐terminus of the protein, depending on which AC domain was attached to the truncated ExsY protein (Figure 11b).

With the N‐terminus AC fusions, strong activity was obtained with all of the combinations of ExsY with ExsY CT (Figure 11c). The ExsY CT protein was, however, not capable of self‐association in this assay. The CotY 119 residue CT protein was not capable of partnering with full‐length CotY or with itself with either the N‐terminus or C‐terminus AC fusions. The failure to associate with full‐length CotY was surprising given the results obtained with ExsY interactions with its CT form and the sequence similarity of the CotY and ExsY CT protein forms. The results were consistent with the CotY CT‐mCherry fusion displaying inefficient incorporation into the exosporium in the *cotY* null mutant spores (Figure 6).

### 3.9 CotY and ExsY protein interactions with CotE and CotO in a bacterial two‐hybrid system

**Figure 12:**
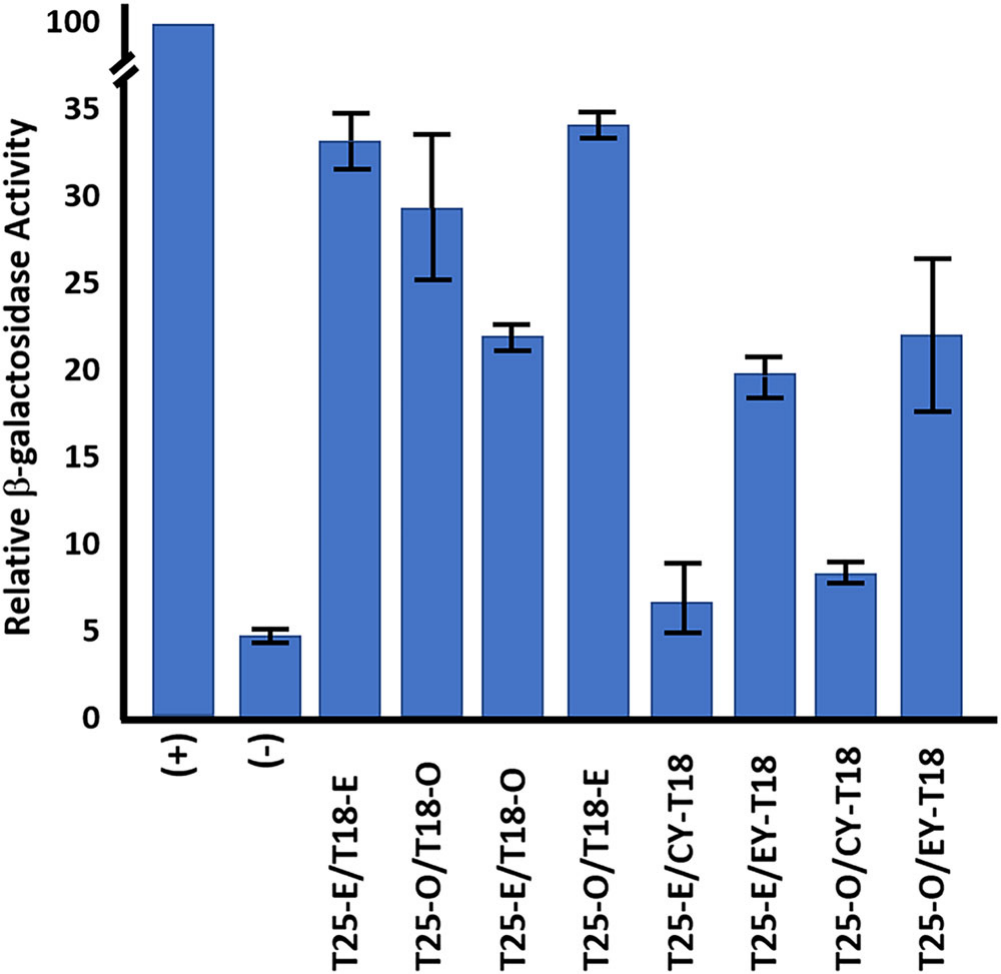
Bacterial two‐hybrid results for CotE (E), CotO (O), ExsY (EY), and CotY (CY). Fusions of the T25 and T18 AC domains to the N‐terminus of the spore proteins are shown. Values are expressed as the percentage of activity of the zip/zip positive control. Values are representative of at least three independent assays and the mean and standard deviation are shown.

During exosporium assembly, the basal layer is anchored to the spore coat by a linkage system that involves the CotE and CotO proteins (Boone et al., 2018; Giorno et al., 2009). Mutants lacking either of these proteins produce an exosporium that fails to associate with the spore surface and is found as sheets at the mother cell central pole of the developing spore. N‐terminus His_6_‐tagged recombinant CotE and CotO proteins were found to interact, with the CotE protein thought to be at the outer spore coat and CotO further toward the exosporium basal layer (Boone et al., 2018). The nature of the interaction of this linkage chain with the exosporium basal layer is unknown. To investigate this, we examined whether interactions among the CotE, CotO, CotY, and ExsY proteins can be determined using the bacterial two‐hybrid approach. The results of this study are shown in Figure 12. Self‐interactions were detected in *E. coli* with the CotE and CotO proteins. Interactions between CotE and CotO were also evident. These results were obtained with N‐terminal AC fusions. No positive protein–protein interactions were detected with the C‐terminal AC domain fusions with either CotE or CotO and so are not included in Figure 12t. CotE and CotO were both able to partner with ExsY and, with lower overall β‐galactosidase activity, with CotY. By means of pull‐down assays, Lablaine et al. (2021) found that CotE can form complexes with CotY and with ExsY during the sporulation of *B. cereus*.

## 4 DISCUSSION

The use of fluorescent protein fusions has proven invaluable in protein localization studies. However, the results of these studies have to be interpreted with caution given the fact that the proteins being studied are altered through the fusion of the reporter protein sequence. This can, in some cases, result in altered folding of the protein, steric hindrance preventing proper interactions with potential partnering proteins or formation of insoluble protein complexes. Such fusion proteins have been employed to sort out details of the exosporium assembly process during sporulation by *B. anthracis* and the closely related species *B. cereus* and *B. thuringiensis*. While the eGFP and mCherry fusion proteins for ExsY and CotY do localize to the predicted sites in the exosporium and in the expected time frame, we demonstrated in this study that the fusion gene constructs are not fully functional and do not complement null mutations of the *exsY* and *cotY* determinants. However, the nature of the defects permitted the identification of intermediate stages in the exosporium basal layer assembly process, which enabled the nature of the fusion protein defect to be determined with some degree of confidence. Expression of the fusion proteins from plasmids yielded similar localization patterns as did the single copy expression system. However, with the plasmid expression, there existed a subset (often substantial) of spores that did not exhibit fluorescence. This was not observed with the single copy expression system. The lack of fluorescence was not the result of plasmid loss during the growth of the bacterial cells or during sporulation. When spores were plated on antibiotic‐free media and the resulting colonies toothpick‐inoculated onto antibiotic‐containing plates, >99% of the colonies exhibited the expected antibiotic resistance.

Timing of expression appears to be the most important feature that results in CotY appearing predominantly at the bottlecap region of the exosporium basal layer and the later expressed ExsY protein predominantly positioned in the noncap portion of the exosporium. Transcription of the *B. thuringiensis exsY* gene occurs with the σ^K^‐bearing RNA polymerase and given the sequence identities, this is likely true with *B. anthracis* (Peng et al., 2016). Transcription of *cotY* has not been studied, but the promoter has a good match to the σ^K^ consensus sequence. Potentially active transcription factors that may impact transcription kinetics have not yet been investigated. Promoters recognized by σ^K^ and the earlier acting σ^E^ have almost identical −10 consensus sequences with the principal difference between the two classes of promoters residing at their −35 regions (Eichenberger et al., 2003). Therefore, some promoters under the control of σ^K^ might also be recognized to some extent by σ^E^. If this is true for the *cotY* promoter, it may explain the earlier initiation of transcription. In prior TEM studies, the cap is the earliest appearing exosporium structure, first evident during the engulfment stage of sporulation (Boone et al., 2018). At this early stage of sporulation, the cap is immature, lacking the electron density and nap layer characteristic of the exosporium of mature spores. CotY, whose gene is transcribed earlier than that of *exsY*, is the major structural component of the cap. CotY is positioned at the mother cell central pole of the spore through interactions with a connector chain with the spore coat. CotE and CotO are known to be components of this linkage structure (Boone et al., 2018; Lablaine et al., 2021). In mutants lacking either CotE or CotO, the cap does not appear, and the exosporium forms but does not assemble around the developing spore, appearing as sheets in the mother cell cytoplasm, adjacent to the cap pole of the spore. Protein–protein interactions between CotE and CotO, and between each of these two proteins and CotY or ExsY were detected in the *E. coli* bacterial two‐hybrid system. The stoichiometry between expression of the linkage chain proteins and that of the exosporium basal layer components is important. Presumed plasmid‐based overexpression of CotE or CotO results in spores lacking an attached exosporium (Boone et al., 2018; Giorno et al., 2007). This presumably results because with the overproduction, some linker chains attach to the outer spore coat and some attach to the exosporium basal layer, but insufficient numbers of these chains are attached to both structures. Plasmid expression of ExsY or CotY does not result in a loss of an exosporium, perhaps because levels of CotY and ExsY are more than adequate for interactions with the CotE‐CotO linker and subsequent cap and noncap self‐assembly. Excess (unincorporated) ExsY and/or CotY proteins would be lost following mother cell lysis.

After the cap forms, noncap assembly initiates and ExsY is incorporated. With mutants lacking ExsY, only the cap structure is formed (Boydston et al., 2006). Extension of the cap into the noncap portion of the spore is likely limited by the amount of CotY produced, as transcript levels of *cotY* decline in the later stages of spore maturation (Bergman et al., 2006 as plotted in Figure 7). In the presence of ExsY, the CotY cap structure provides a template for the addition of ExsY monomers to create the noncap basal layer with assembly extending toward the noncap pole of the spore. The *cotY* null mutants do not form the cap structure early in sporulation (Boone et al., 2018), but do form an attached exosporium. Boone et al. (2018) provided an explanation for this. In the absence of the cap, an exosporium can form, but with delayed kinetics. ExsY can attach to the linkage chain in the interspace region and once an ExsY patch appears, it can prime further assembly of ExsY around the circumference of the spore to form a complete exosporium. In this case, the assembly initiates most frequently on the lateral spore surfaces, rather than the less surface prominent poles. In this study, both the CotY and ExsY N‐terminus sequences were shown to be capable of positioning the fluorescent reporter around the spore surface. It is tempting to speculate that this N‐terminus sequence is the target for interactions with the CotE/CotO attachment chain. The degenerate nature of the N‐terminus sequence of ExsY may result in a weaker interaction with the attachment chain and thus a longer lag in the initiation of exosporium assembly, as observed with the *cotY* mutant sporulating cells (Boone et al., 2018). The N‐terminus sequence defects in ExsY, in addition to the later transcription of the *exsY* determinant, may also contribute to the preponderance of CotY in the cap structure.

Through the elegant studies of Terry et al. (2017), it was determined that ExsY, and to a lesser extent CotY, can self‐assemble. ExsY forms a two‐dimensional lattice that is stabilized by disulfide linkages and electron crystallography analysis indicating the presence of the same overall structural features of an intact exosporium. For self‐assembly to occur, the protein monomers must be able to recognize other subunits, be correctly positioned, and ultimately stabilized by disulfide bond formation. Our studies with ExsY‐mCherry indicated that this fusion protein can be incorporated into the exosporium basal layer, but cannot serve as a substrate for subsequent monomer incorporation. The mCherry‐ExsY protein, however, fails to incorporate at detectable levels in the presence of ExsY and poorly incorporates in the absence of the unfused protein. This suggests a defect in the addition of the N‐terminus fusion protein to a growing ExsY sheet, possibly through steric hindrance. Surprisingly, the AC fusions at the N‐terminus of both ExsY and CotY did not weaken or prevent protein–protein interactions in the bacterial two‐hybrid assays. It is possible that although the three proteins fused (mCherry, AC T25, and AC T18) are not substantially different in mass (26,722, 18,951, and 24,017 Da, respectively), differences in secondary structure could impact steric effects. Alternatively, the putative steric effect may not be on the capacity of the fusion protein to initially interact but prevents the proper alignment needed for the formation of the stabilizing disulfide bonds. This latter step would not be needed for adenylate cyclase activity in *E. coli* but would be required for the proper positional fixing of the protein in the exosporium sheet structure.

The short CotY and ExsY N‐terminus sequences were sufficient to position the mCherry reporter around the spore, even in mutants that produce only the cap portion of the exosporium or lack the exosporium entirely. This interaction likely results from interactions with proteins within the interspace layer of the spore. The NT proteins do not measurably interact with the CotY or ExsY proteins. On the other hand, the absence of the NT sequences from the ExsY CT protein did not disrupt interactions with ExsY or CotY, although ExsY CT self‐interactions did not demonstrably occur. Thus, ExsY self‐assembly requires the NT sequences. Despite the high sequence identity of the CotY CT and ExsY CT proteins, the CotY CT protein failed to interact with the full‐length CotY protein, unlike the situation observed with ExsY CT/ExsY pairs. CotY CT‐mCherry, however, can interact with full‐length CotY protein, as evident by the cap labeling in the *exsY* null mutant spores. This labeling, however, was relatively weak, suggestive of weaker interactions.

**Figure 13:**
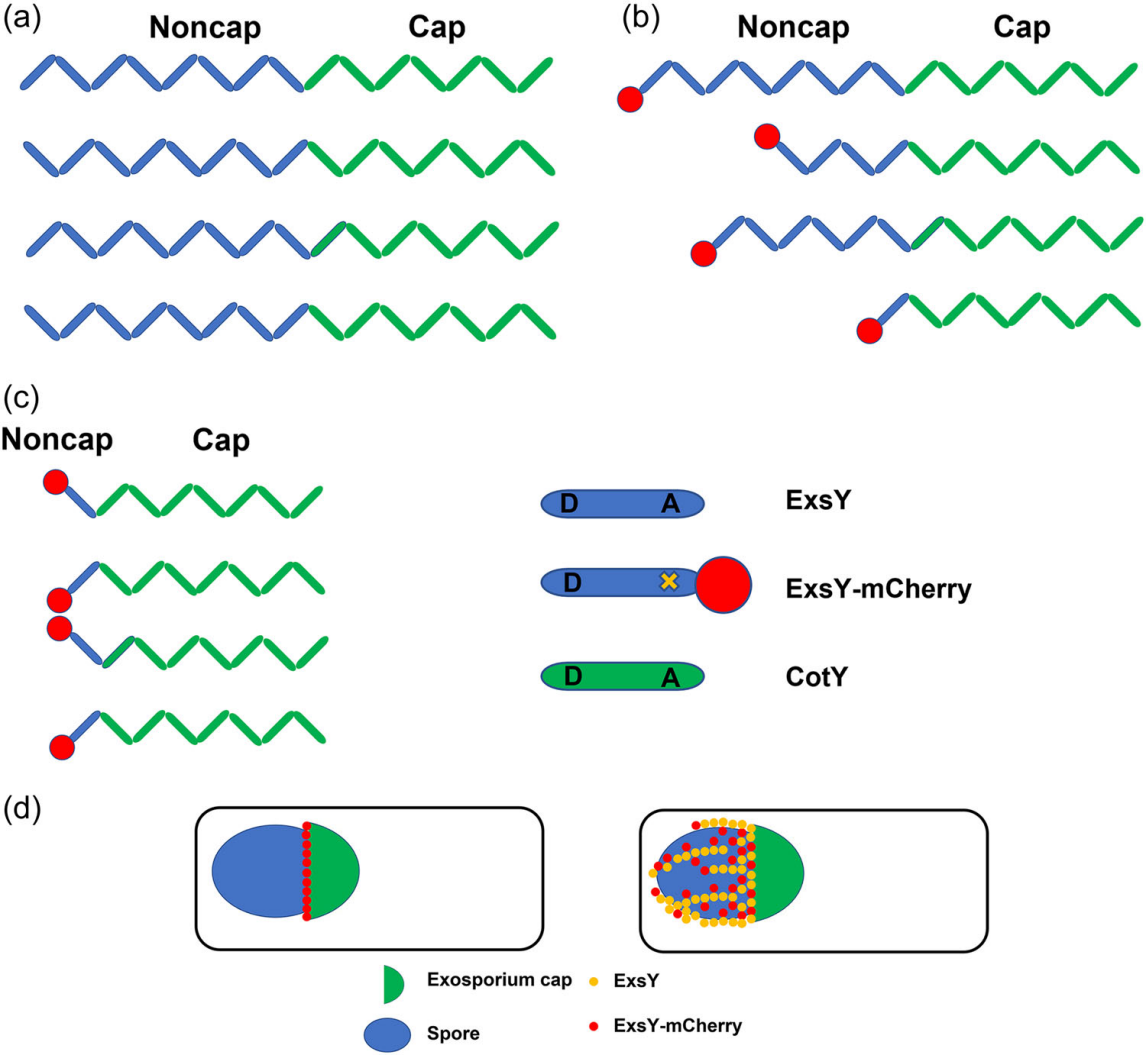
A simplistic view of the CotY and ExsY interactions and the effects of the mCherry fusion on the C‐terminus of ExsY on assembly. The number of partners for CotY or ExsY interactions is currently unknown, so the donor sites (d) and acceptor sites (a) on the proteins do not imply that there may be more than one interactive partner at each site. CotY cap sites are in green and ExsY predominant noncap exosporium is in blue (yellow in panel d). The mCherry fusion is denoted as a red sphere. Panel (a) represents basal layer assembly in Sterne. Panel (b) represents the assembly termination of chain extension resulting from incorporation of ExsY‐mCherry in the noncap. Panel (c) shows the effect of ExsY‐mCherry in an *exsY* null mutant where ExsY‐mCherry can partner with CotY but cannot be extended further. Panel (d) shows the exosporium assembly pattern in sporulating cells expressing ExsY‐mCherry in the absence of ExsY (left) where only the monolayer fluorescent ring is evident and in the presence of ExsY (right) in which case ExsY‐ExsY extension can occur until an ExsY‐mCherry subunit is added and blocks further extension. Because the extension occurs at different stages of noncap assembly, fluorescent labeling occurs throughout the noncap portion of the exosporium.

In our model of exosporium assembly (Figure 13), the initial interaction for CotY would be with the CotE/CotO containing linker at the mother cell‐central pole of the developing spore, and following this positioning event, CotY‐CotY interactions then form the cap structure. The initial interaction site for an incoming monomer involves the N‐terminus sequences (which we call the donor site in the model). The donor site interacts with an acceptor site, likely located within the larger CT portion of the proteins. The margin of the cap portion of the exosporium inner basal layer provides the site of initial attachment of ExsY and the subsequent addition of ExsY forms the noncap basal layer with assembly proceeding from the cap to the noncap pole. The plasmid‐based expression of the CotY‐ and ExsY‐fusion proteins suggest that ExsY can be incorporated into the cap and CotY into the noncap, but the extent that this occurs under normal cellular levels of these proteins is unknown. Incorporation of ExsY‐mCherry or CotY‐mCherry can occur (functional donor site) but this acts as a chain termination event (inactive acceptor site). In an *exsY* null mutant background, a monolayer of ExsY‐mCherry is added to the CotY in the cap, producing a ring of fluorescence. In the Sterne host, the presence of unfused ExsY, which is likely more efficiently incorporated relative to the fusion protein, the fusion protein gets incorporated at various stages of noncap assembly, introducing the fluorescent label at different stages of the process and giving a noncap fluorescence pattern (Figure 13d). In Sterne hosts, CotY‐mCherry can interact with CotY to label the cap. With the *cotY* null mutant, the cap structure does not form early in the sporulation process (Boone et al., 2018). The CotY‐mCherry protein cannot form a cap (defective acceptor site) in this background. When ExsY eventually primes exosporium assembly, the CotY‐mCherry protein can pair with ExsY and get incorporated, resulting in the observed exosporium fluorescence. Presumably, the fusion protein is less efficiently incorporated relative to unfused ExsY, thus permitting the completion of the exosporium assembly.

This overly simplistic model functionally implies a donor site and acceptor site on the ExsY and CotY proteins. However, there may be more sites of interaction on these proteins. The tagging of CotY or ExsY at their carboxyl terminus with the fluorescent protein, however, functionally blocks interactions with additional protein subunits, both unfused and fused. Fusions with mCherry or eGFP at the amino terminus of CotY or ExsY substantially interfere with incorporation. The bacterial two‐hybrid results suggest this is not due to preventing pairing of the proteins, but may prevent effective pairing that leads to disulfide bond formation and thus stable incorporation. However, the interactions must be weak, given that the ExsY‐mCherry protein was shown to assemble around the spore whereas the mCherry‐ExsY protein remained in the cytoplasm of late‐stage sporulating cells (Figure 4). With Sterne, the unfused CotY and ExsY proteins thus outcompete the fusion proteins for incorporation. The mCherry‐ExsY fusion protein, expressed in the *exsY* null host, has no ExsY to compete with, which results in labeling at the CotY‐containing bottlecap margin.

Following synthesis of the basal layer, the size of the interspace increases, possibly due to cleavage of the CotE/CotO chain that positioned the exosporium during the assembly process (Boone et al., 2018). Possible evidence for such a proteolytic event was the finding of an N‐terminal CotE peptide (residues 2–14) in an exosporium proteomic study (Todd et al., 2003).

## AUTHOR CONTRIBUTIONS

**Jorge Durand‐Heredia**: Formal analysis (equal); Investigation (equal); Methodology (equal); Writing – review and editing (equal). **George C Stewart**: Conceptualization (lead); Data curation (equal); Formal analysis (equal); Funding acquisition (lead); Investigation (equal); Project administration (lead); Resources (lead); Supervision (lead); Writing – original draft (lead); Writing – review and editing (equal).

## CONFLICT OF INTEREST

None declared.

## ETHICS STATEMENT

None required.

## Data Availability

All data are provided in full in this paper.

## 1

**Table A1:** Bacterial strains and plasmids used in this study

Strain	Relevant characteristic(s)	Source or reference
***Bacillus anthracis***		
MUS8228	Sterne *ΔexsY*	This study
MUS8229	Sterne *ΔcotY*	This study
MUS8230	Sterne *ΔcotY ΔexsY*	This study
MUS8233	Sterne *ΔamyS::exsY*	This study
MUS8234	Sterne *ΔamyS::exsY ‐ mcherry*	This study
MUS8235	Sterne *ΔamyS::cotY ‐ egfp exsY ‐ mcherry*	This study
Sterne	pXO1^+^ pXO2^−^	Lab stock
***E. coli***		
BTH101	*cya‐99, rpsL1 (*Str^r^ *), hsdR2, mcrA1, mcrB1*	Karimova et al. (2017)
DH5α	*φ80dlacZΔM15 recA1 endA1 gyrA96 thi‐1 hsdR17*	Lab stock
	*(r*_*K*_^*−*^ *m*_*K*_^*−*^ *) supE44 relA1 deoR Δ(lacZYA‐argF)U169*	
GM48	*F* ^*–*^, *thr, leu, thi, lacY galK galT ara fhuA tsx dam*	Lab stock
	*dcm glnV44*	
**Plasmids**		
**Plasmid**	**Description**	**Source or reference**
pGS4080	pSI‐1 *cre*	This study
pDG4099	pMK4 *egfp* fusion vector	Boone et al. (2018)
pDG4100	pMK4 *mcherry* fusion vector	Hermanas et al. (2021)
pGS4294	Allele replacement ts shuttle vector Amp^r^, Ery^r^	Lab stock
pGS4652	pMK4‐*exsY‐egfp*	This study
pGS4709	pMK4 *bclA* promoter +rbs ‐ *mcherry*	This study
pGS6272	pMK4‐P_*exsY*_‐*egfp‐exsY*	This study
pGS6274	pMK4‐P_*exsY*_‐*mcherry‐exsY*	This study
pGS6275	pMK4‐P_*cotY*_‐*mcherry‐cotY*	This study
pGS6328	pGS4295‐*amyS::spc*; Amp^R^, Ery^R^, Spc^R^ ts	This study
	shuttle vector	
pGS6276	pMK4‐P_*cotY*_–*egfp‐cotY*	This study
pHPS2	Shuttle vector; Spc^r^, Ery^r^	Thompson et al. (2011)
pJD4962	pHPS2‐*exsY‐egfp*	This study
pJD4963	pHPS2‐*cotY‐mcherry*	This study
pJD4964	pHPS2‐*cotY‐egfp*	This study
pJD6116	pHPS2‐P_*exsY*_ *‐exsY NT*‐*mcherry*	This study
pJD6117	pHPS2‐P_*cotY*_ *‐cotY NT*‐*mcherry*	This study
pJD6140	pHPS2‐P_*exsY*_ *‐cotY NT‐exsY CT‐mcherry*	This study
pJD6164	pHPS2‐P_*exsY*_ *‐cotY‐mcherry*	This study
pJD6165	pHPS2‐P_*cotY*_ *‐exsY NT‐cotY CT‐mcherry*	This study
pJD6174	pHPS2‐*exsY*	This study
pJD6270	pHPS2‐*exsY‐mcherry*	This study
pJD6430	pHPS2‐P_*cotY*_ *‐exsY‐mcherry*	This study
pJD6431	pHPS2‐P_*exsY*_ *‐exsY CT‐mcherry*	This study
pJD6432	pHPS2‐P_*cotY*_ *‐cotY CT‐mcherry*	This study
pJD6433	pGS6328 *exsY‐mcherry*	This study
pJD6434	pGS6328‐*cotY‐egfp‐t*_*cotY*_ *‐exsY‐mcherry*	This study
pJD6435	pGS6328 P_*cotY*_ *‐exsY‐mcherry*	This study
pJD6436	pGS6328 P_*exsY*_ *‐cotY‐mcherry*	This study
pJD6437	pHPS2‐P_*cotY*_ *‐exsY‐egfp*	This study
pJD6438	pHPS2‐P_*cotY*_ *‐exsY‐mcherry*	This study
pJD6439	pHPS2‐P_*exsY*_ *‐cotY‐egfp*	This study
pJD6440	pHPS2‐P1_*exsY*_ *‐exsY‐mcherry*	This study
pJD6441	pHPS2‐P2_*exsY*_ *‐exsY‐mcherry*	This study
pJD6442	pKT25‐T25‐*aw20_5669*	This study
pJD6443	pKT25‐T25‐*exsY*	This study
pJD6444	pKT25‐*T25*‐*cotY*	This study
pJD6445	pKNT25‐*exsY‐T25*	This study
pJD6446	pUT18‐*exsY–T18*	This study
pJD6447	pKNT25‐*exsY NT*–*T25*	This study
pJD6448	pUT18 ‐ *exsY NT–T18*	This study
pJD6449	pKNT25‐*exsY CT–T25*	This study
pJD6450	pUT18‐*exsY CT–T18*	This study
pJD6451	pKNT25‐*cotY‐T18*	This study
pJD6452	pUT18‐*cotY‐T18*	This study
pJD6453	pKNT25‐*cotY NT‐T25*	This study
pJD6454	pUT18‐*cotY NT‐T18*	This study
pJD6455	pKNT25‐*cotY CT‐T25*	This study
pJD6456	pUT18‐*cotY CT‐T18*	This study
pJD6457	pUT18C‐*T18*‐e*xsY*	This study
pJD6458	pKT25‐*T25*‐*exsY NT*	This study
pJD6459	pUT18C‐*T18‐exsY NT*	This study
pJD6460	pKT25‐*T25‐exsY CT*	This study
pJD6461	pUT18C‐*T18‐exsY CT*	This study
pJD6462	pUT18C‐*T18‐cotY*	This study
pJD6463	pKT25‐*T25‐cotY NT*	This study
pJD6464	pUT18C‐*T18‐cotY NT*	This study
pJD6465	pKT25‐*T25‐cotY CT*	This study
pJD6466	pUT18C‐*T18‐cotY CT*	This study
pJD6467	pKT25‐*T25‐cotE*	This study
pJD6468	pUT18C‐*T18‐cotE*	This study
pJD6469	pKT25‐*T25‐cotO*	This study
pJD6470	pUT18C‐*T18‐cotO*	This study
pKNT25	Kan^r^, in‐frame fusions at the C‐terminal end of	Karimova et al. (2017)
	the T25 adenylate cyclase polypeptide	
pKT25	Kan^r^, in‐frame fusions at the N‐terminal end of	Karimova et al. (2017)
	the T25 adenylate cyclase polypeptide	
pKT25‐Zip	pKT25 with Zip domain	Karimova et al. (2017)
pMK4	Shuttle plasmid Amp^r^, Cm^r^	Sullivan et al. (1984)
pSI‐1	Shuttle plasmid; IPTG inducible; Cm^R^	Lee et al. (2003)
pUT18	Amp^r^; in‐frame fusions at the N‐terminal end of	Karimova et al. (2017)
	the T18adenylate cyclase polypeptide	
pUT18C	Amp^r^; in‐frame fusions at the C‐terminal end of	Karimova et al. (2017)
	the T18adenylate cyclase polypeptide	
pUT18C‐Zip	pUT18C with Zip domain	Karimova et al. (2017)

*Note*: Amp^r^, Cm^r^, Ery^r^, Kan^r^ and Spc^r^ denote resistance to 100 μg ampicillin ml^−1^, 10 μg chloramphenicol ml^−1^, 5 μg erythromycin ml^−1^, 50 μg kanamycin ml^−1^, and 100 μg spectinomycin ml^−1^, respectively.
